# The structure dilemma in biological and artificial neural networks

**DOI:** 10.1038/s41598-021-84813-6

**Published:** 2021-03-10

**Authors:** Thomas Pircher, Bianca Pircher, Eberhard Schlücker, Andreas Feigenspan

**Affiliations:** 1 Institute of Process Machinery and Systems Engineering, Friedrich-Alexander University Erlangen-Nuremberg, Cauerstraße 4, 91058 Erlangen, Germany; 2 Department Biology, Animal Physiology, Friedrich-Alexander University Erlangen-Nuremberg, Staudtstraße 5, 91058 Erlangen, Germany

**Keywords:** Network models, Network models, Machine learning

## Abstract

Brain research up to date has revealed that structure and function are highly related. Thus, for example, studies have repeatedly shown that the brains of patients suffering from schizophrenia or other diseases have a different connectome compared to healthy people. Apart from stochastic processes, however, an inherent logic describing how neurons connect to each other has not yet been identified. We revisited this structural dilemma by comparing and analyzing artificial and biological-based neural networks. Namely, we used feed-forward and recurrent artificial neural networks as well as networks based on the structure of the micro-connectome of *C. elegans* and of the human macro-connectome. We trained these diverse networks, which markedly differ in their architecture, initialization and pruning technique, and we found remarkable parallels between biological-based and artificial neural networks, as we were additionally able to show that the dilemma is also present in artificial neural networks. Our findings show that structure contains all the information, but that this structure is not exclusive. Indeed, the same structure was able to solve completely different problems with only minimal adjustments. We particularly put interest on the influence of weights and the neuron offset value, as they show a different adaption behaviour. Our findings open up new questions in the fields of artificial and biological information processing research.

## Introduction

Going far beyond the obvious macroscopic structure of the brain, which hardly differs between human individuals, various authors postulated a coupling of structure and function^1–3^. Neurons, hubs, or in graph theory called nodes, with similar connection patterns often show similar functionality^4,5^. On the contrary, several studies have repeatedly shown that the brains of patients suffering from schizophrenia or other neurological diseases, have a different connectome than healthy people^6,7^. This is particularly evident in patients suffering from severe stroke, other lesions within the central nervous system or after a massive hemispherectomy, in which the entire right hemisphere has been removed. Despite these damages, several patients have been able to learn to speak again, and they acquired all language-related abilities^8^. Although their brains showed an altered structure, it included strongly preserved parts of the initial brain regions responsible for language processing^8,9^.

Nevertheless, and this inevitably leads to a highly discussed paradox and known as the structure dilemma^2^, it must be acknowledged that this structure is not exclusive. There is no ‘master plan’ of the brain, no fixed wiring diagram, nor a completely determined structure defined in each individual’s genetic code. While genetics ensures that essential, mainly macroscopic, structures develop in an appropriate spatiotemporal pattern, subsequent refining steps, as the wiring between individual neurons, are thought to be influenced to a large extent by randomness. Early anatomical studies of different brain regions support the notion of a high degree of randomness during network formation in the developing brain^10–13^ and the ongoing learning process in the adult brain^5^. Braitenberg and Schüz even describe the cortex as a ‘mixing device’, whose connections are set up in a largely random manner^14^. Probably the most spectacular example of a healthy human brain is that of a 44 year-old man with a massive ventricular enlargement resulting in an grossly altered structure of the entire brain^15^.

**Figure 1:**
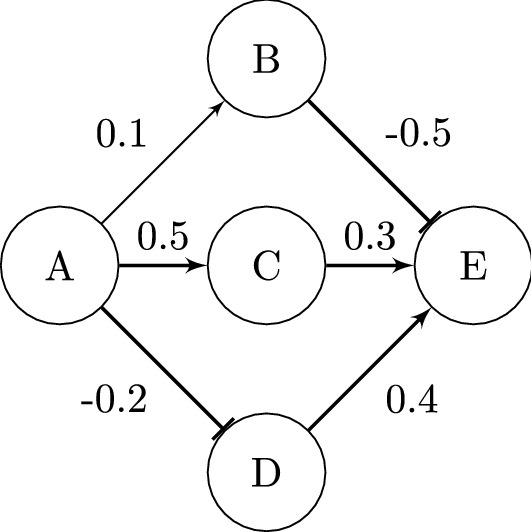
Schematic of converting a dense network into a sparse graph by choosing the five strongest edges. Weak edges are interpreted as a not existing connection (A $\nrightarrow$ B). Positive weights are activating (A $\rightarrow$ C, C $\rightarrow$ E, D $\rightarrow$ E) and negative weights are inhibiting (A $\dashv$ D, B $\dashv$ E) connections.

Indeed, also artificial neural networks (ANNs) have revealed similar representations for both sides of the dilemma^16^. On the one hand, calculations with ANNs, as for example random initialization methods, suggest a high degree of randomness, but it has also been found that subnets or, in other words, a pre-set structure, take over problem solving^17,18^. These subnetworks, in this recent publication described as *winning tickets*^17^, ‘win the initialization lottery’, as their initial weights were able to solve problems with unsurpassed accuracy. Since artificial neural networks are confronted with ever more complex problems, whereas computing power is not unlimited, techniques to reduce the general network size have been developed^19,20^. An elimination of unnecessary weights from a neural network is called *pruning* and indeed, it has been shown that the network size can be reduced by more than 90$\%$ without a significant loss of accuracy^17,21,22^. These phenomena are based on the findings that not all parts of a network are equally important. Based on these ideas, we considered these sparse (in contrast to dense networks, in which every neuron is connected to each other) artificial neural networks as graphs and separated the raw structure. That means that the connections within the network are only defined as activating, inhibiting or non-existing (illustrated in Fig. 1). Consequently, in this paper the ‘structure’ always refers to the specific connections within these sparse networks and therefore is considered as the graph theoretical connectome of the artificial neural network. The biological connectome was likewise described and analysed as a graph^2,23–25^, which allows a comparison and analysis of both network structures.

The aim of this paper is to compare information processing in biological neural networks with that in ANNs, to get a deeper insight into the structural dilemma of both, types of neural networks. For a valid comparison and an interdisciplinary approach we carried out diverse experiments with artificial neural networks, including two feed-forward and two recurrent architectures, as well as two biologically-based neural networks. As biological networks we used the wiring diagram of the male *C. elegans*^26^, as the only living organism whose neural system has been mapped in its entirety, representing the micro-connectome level, and second, the functional *connectome* data of healthy patients representing the macro-connectome level^27^. Experiments for all networks were performed with different initialization and pruning methods, on different training sets, as well as with disturbed or blocked weights and bias values during the training process. The bias value is a parameter in the calculations of artificial neural networks that provides an additive offset for each neuron to adjust its sensitivity to its activation. To avoid any misunderstandings in the context of the word bias, we will refer to it as the ‘neuron offset value’.

Through this systematic analysis we wanted to gain a deeper insight into the relevance of the structure for information processing and to understand the dilemma from a technical perspective.

## Results

### Structure contains the information

**Figure 2:**
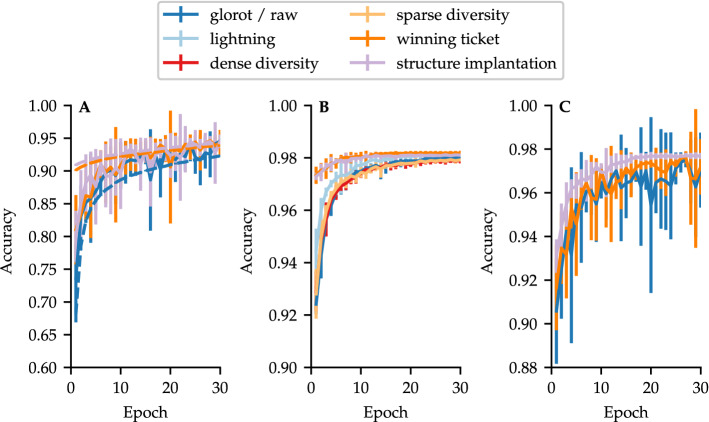
Learning curves showing the validation accuracy of different networks over 30 epochs of learning with different initialization methods for the *MNIST* problem. Error bars show standard deviation over ten independent random iterations. (**A**) *C. elegans* biological-based recurrent (solid lines) and feed forward network (dashed lines). (**B**) Feed forward network *LeNet 4x300*. (**C**) *G*(*n*, *p*) *1194.*

We mentioned in the introduction that in biological systems the neuronal structure apparently contributes considerably to information processing. When transferring this approach to artificial neural networks, three questions arise: (1) how much information does the structure of an ANN contain related to the weights, (2) does a functional biological network with its distinct structure can be translated in an artificial neural network, and (3) which changes arise in relation to learning performance and characteristics, when computing these technically transferred networks? To answer these questions we performed several experiments and tried to implement biological connectomes as artificial neural networks. In the following we will refer to them as biological-based neural networks (BBNN). For this purpose, we first used the micro-connectome of chemical connections of *C. elegans*^26^ and transformed it in a recurrent and a feed-forward architecture with similar distributed path lengths (see “Methods” for a detailed description). Indeed, as the learning curves in Fig. 2A shows, the raw structure of the nematode neurons were also able to learn like an ANN. The feed-forward configuration is printed in dashed lines, whereas the solid lines show the recurrent architecture. The feed-forward architecture reaches an accuracy of approximately 90$\%$, whereas the recurrent configuration reaches 93$\%$ accuracy after 30 epochs of learning.

In addition, we initialized the network with either *winning ticket*^17^ (orange) or *structure implantation* (purple) to investigate whether the pre-trained structure contains enough or the same information as the weights themselves. In contrast to the *winning ticket* initialization, in which this structure including its weights has been implanted, the *structure implantation* only initialized the structure with fixed values for the categories exciting, inhibiting, and no connections (explained in Fig. 1), but without any distinct information about the weights. Despite the fact that weight information was not provided, both *C. elegans* configurations showed approximately the same accuracy (95$\%$ in the recurrent architecture), whereas the faster approach was particularly pronounced in the feed-forward architecture. This result is also obtained for the commonly used *LeNet 4x300* (see Fig. 2B). Here, the faster approximation of a learned state, as the gradient of the accuracy became small and there was no improvement in subsequent learning epochs, is noteworthy. Other initialization methods as *dense* or *sparse diversity* (see “Methods”) did not show significant improvements in relation to the random *glorot* initialization (best case of learn curve sum $\mathrm {max}~t=1.11$ with $\mathrm {min}~p=0.14$). The significance level is $5\%$. A minor optimisation was obtained by *lightning*^28^, which outperforms the other random initialization methods (worst case of learn curve sum $\mathrm {min}~t=6.75$ with $\mathrm {max}~p=1.26 \cdot 10^{-6}$). Figure 2C shows the learning curve of a recurrent network in a random *G*(*n*, *p*) configuration with 1194 nodes. Although all three learning methods show approximately the same behaviour and final result, both *winning ticket* and *raw* display, in contrast to the *structure implantation* great fluctuations in their accuracy during learning iterations. This may be caused by a possible restriction due to the specification of too concrete weights, which could be exactly the wrong ones for this particular learning process state. It seems that an initialization with only the structure and no weight information is more successful. Therefore, it can be stated that the structure already contains all needed information.

**Figure 3:**
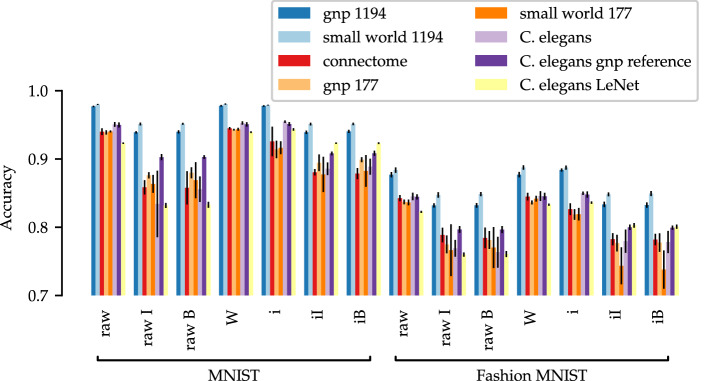
Best accuracy of the recurrent experiments. Error bars show standard deviation over ten independent random iterations. I—iterative pruning, B—bio pruning; W—winning ticket, i—structure implantation.

To examine this in more detail, we trained eight different networks on two training data sets with different initialization and pruning methods and compared them by their best accuracy (Fig. 3). In particular, we used recurrent networks with 1194 and 177 nodes in a random *G*(*n*, *p*) configuration or with a small world architecture, respectively (the uniform and IRNN configurations are shown in the Supplementary Material). This decision was based on recent findings in biological and artificial neural networks. Brain networks indeed show the defined high clustering and short path length topology^29^ as demonstrated by various neuroimaging studies^30^ and calculations^31^. Also in a technical context, it has been shown that in feed-forward networks a small world architecture positively influences learning time and error probability^32–35^. In addition, we calculated two different BBNNs. First,the naturally occurring micro-connectome of *C. elegans*^26^, as well as in the previously described recurrent and its feed-forward configuration; and second, technically transferred human *connectomes*^36^ (the 177 node recurrent networks were the corresponding random reference).

Leading in the comparison of the best accuracy of recurrent networks were the networks with 1194 nodes, whereby the small world architecture performed even better (worst not alpha value inflation endangered case for *MNIST* $\mathrm {min}~t=5.16$ with $\mathrm {max}~p=3.25 \cdot 10^{-5}$; worst not alpha value inflation endangered case for *Fashion-MNIST* $\mathrm {min}~t=6.61$ with $\mathrm {max}~p=1.67 \cdot 10^{-6}$). The alpha inflation endangered cases are listed in Table 1, which based on $m = 88$ data accesses and the Bonferroni correction $p \le \frac{\alpha }{m} = 0.057\%$. These findings seem to put into perspective previous results of advantages of a network with small world architecture^32,33^. However, it should be kept in mind that we have only considered recurrent networks, but not the modified version of a feed-forward network, as it was used in e. p.^34^. The size of the network must also be taken into account here. The effect of a considerable improvement only occurred in larger networks. In the smaller 177 node configuration no consistent behavior in the accuracy could be observed as shown in Table 2, whereby here pruning had strong negative effects on the accuracy. The profitable benefits of this specialized structure seem to be cancelled out if the computing capacity is not large enough. Table 3 shows additionally the statistical t-test results on the best accuracy and the learn curves sum for the different architectures. In general *structure implantation* and *winning ticket* perform similar. *Structure implantation* performs better related to the learn curve sum, excepted for the small 177 architectures. In some cases *winning ticket* is better than *structure implantation* related to the best accuracy, but the differences are very small. The values are only definite for the small random networks (*G*(*n*, *p*) *177* and *small world 177*) where *winning ticket* is clearly better.

**Table 1:** Endangered by alpha value inflation accuracy t-test for ‘*small world 1194* is best’.

Data set	Experiment	*t*	*p* (%)
*MNIST*	i	3.02	$0.37$
*Fashion-MNIST*	Raw	3.84	$0.06$
	i	2.88	$0.50$

i—structure implantation.

**Table 2:** Accuracy t-test for ‘*small world 177* is better than *G*(*n*, *p*) *177*’.

Data set	Experiment	*t*	*p* (%)
*MNIST*	Raw	1.34	$9.91$
	Raw I	− 2.88	$0.50$
	Raw B	− 1.27	$11.4$
	W	0.81	$21.5$
	i	0.49	$31.4$
	iI	− 1.88	$3.79$
	iB	2.22	$1.96$
*Fashion-MNIST*	Raw	− 0.43	$33.5$
	Raw I	− 0.67	$25.5$
	Raw B	− 1.06	$15.1$
	W	3.31	$0.20$
	i	0.15	$44.2$
	iI	− 3.53	$0.12$
	iB	− 4.02	$0.04$

I—iterative pruning, B—bio pruning; W—winning ticket, i—structure implantation.

**Table 3:** Accuracy and learn curve sum t-test for ‘*structure implantation* is better than *winning ticket*’ (negative values indicating *winning ticket* is better than *structure implantation*).

Data set	Architecture	Best accuracy			Learn curve sum	
		Difference (%)	*t*	*p* (%)	*t*	*p* (%)
MNIST	*G*(*n*, *p*) *1194*	$-0.03$	− 0.62	$27.1$	6.53	$0.00$
	*Small world 1194*	$-0.14$	− 4.20	$0.03$	1.12	$13.8$
	*G*(*n*, *p*) *177*	$-2.90$	− 7.09	$0.00$	− 3.84	$0.06$
	*Small world 177*	$-2.71$	− 8.83	$0.00$	− 7.74	$0.00$
	*Connectome*	$-0.05$	− 0.32	$37.8$	1.88	$3.80$
	*C. elegans* *G*(*n*, *p*)	$0.05$	0.34	$36.7$	2.22	$1.99$
	*C. elegans LeNet*	$0.41$	5.89	$0.00$	9.25	$0.00$
	*C. elegans*	$0.19$	1.92	$3.52$	− 0.15	$44.1$
Fashion- MNIST	*G*(*n*, *p*) *1194*	$0.68$	4.73	$0.01$	4.69	$0.01$
	*Small world 1194*	$-0.02$	− 0.13	$44.9$	1.81	$4.33$
	*G*(*n*, *p*) *177*	$-1.77$	− 6.71	$0.00\%$	− 8.93	$0.00$
	*Small world 177*	$-2.26$	− 7.08	$0.00$	− 8.34	$0.00$
	*Connectome*	$0.13$	0.76	$22.7$	1.79	$4.51$
	*C. elegans* *G*(*n*, *p*)	$0.26$	1.19	$12.4$	0.11	$45.6$
	*C. elegans LeNet*	$0.30$	3.97	$0.05$	5.93	$0.00$
	*C. elegans*	$0.41$	1.75	$4.84$	1.85	$4.01$

Another argument supporting this hypothesis concerns the results of *C. elegans*. In the ranking of the best accuracy, *C. elegans* in its unpruned configurations can be positioned directly after the 1194 nets (worst not alpha value inflation endangered significant case for *MNIST* $\mathrm {min}~t=7.88$ with $\mathrm {max}~p=1.52 \cdot 10^{-7}$; worst significant case for *Fashion-MNIST* $\mathrm {min}~t=3.96$ with $\mathrm {max}~p=4.64 \cdot 10^{-4}$). Table 4 shows the cases that are not significant. The unrestricted network showed the best results (worst case for *MNIST* $\mathrm {min}~t=7.54$ with $\mathrm {max}~p=2.80 \cdot 10^{-7}$; worst case for *Fashion-MNIST* $\mathrm {min}~t=11.0$ with $\mathrm {max}~p=1.02 \cdot 10^{-9}$), whereas in the pruning experiments without pre-trained initialization it displayed the largest decline (worst not alpha value inflation endangered case for *MNIST* $\mathrm {min}~t=4.55$ with $\mathrm {max}~p=1.24 \cdot 10^{-4}$; worst significant case for *Fashion-MNIST* $\mathrm {min}~t=11.0$ with $\mathrm {max}~p=1.09 \cdot 10^{-9}$). Table 5 shows the not significant cases. These results are understandable since the neural structure of *C. elegans* was not optimised to solve the *MNIST* problem. Neurobiological research postulated that a specialized structure is always coupled with a certain function^2,3^. However, the structure needs to be changed only slightly so that it can solve this synthetic task more effectively, as evident in comparison with the *C. elegans* *G*(*n*, *p*) *reference* variant (not significant cases are shown in Table 4). The use of the pre-trained information of *structure implantation* improves the performance for the raw *C. elegans*, thereby emphasizing the argument of only minimal changes as shown in Table 6.

**Table 4:** Exceptions and alpha value inflation endangered cases for the accuracy t-test for ‘*C. elegans* is the third best’.

Data set	Architecture	Experiment	*t*	*p* (%)
*MNIST*	*C. elegans* *G*(*n*, *p*) *reference*	Raw	0.37	$35.8$
		W	1.53	$7.23$
		i	3.18	$0.26$
*Fashion-MNIST*	*G*(*n*, *p*) *177*	W	3.85	$0.06$
	*Small world 177*	W	1.50	$7.54$
	*C. elegans* *G*(*n*, *p*) *reference*	Raw	0.29	$38.7$
		W	0.13	$45.0$
		i	1.18	$12.6$

W—winning ticket, i—structure implantation.

**Table 5:** Exceptions and alpha value inflation endangered cases for the accuracy t-test for largest decline for not pre-trained pruning by *C. elegans*.

Data set	Pre-trained	Not pre-trained	*t*	*p* (%)
*MNIST*	iI	Raw I	3.30	$0.20$
	iB	Raw I	3.45	$0.14$
*Fashion-MNIST*	iI	Raw I	1.57	$6.68$
	iB	Raw I	1.44	$8.37$
	iI	Raw B	1.80	$4.41$
	iB	Raw B	1.70	$5.32$

I—iterative pruning, B—bio pruning; i—structure implantation.

**Table 6:** Accuracy t-test for improvement through pre-trained information for *C. elegans*.

Data set	Pre-training	*t*	*p* (%)
*MNIST*	W	1.60	$6.35$
	i	3.24	$0.22$
*Fashion-MNIST*	W	0.19	$42.4$
	i	2.49	$5.32$

W—winning ticket, i—structure implantation.

**Figure 4:**
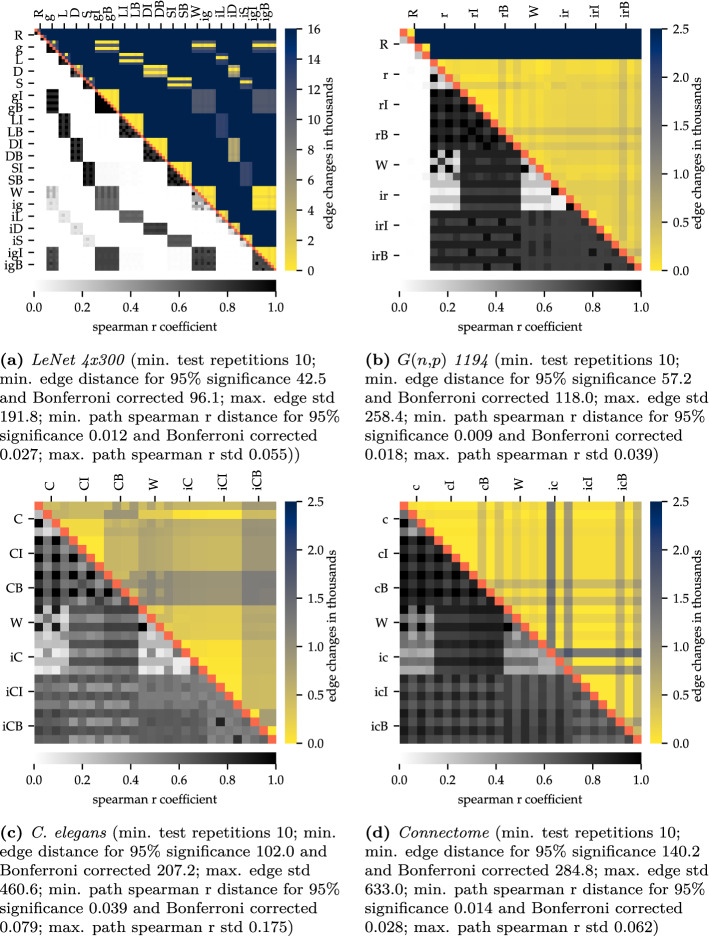
Toast plot type 1: Edge and weight changes during learning and between different initial states. Identical tests marked red. Each run is marked with an abbreviation at the side of the graph. Each of these include four different subvariants (each as its own pixel), entailing the initial status or the trained status after 30 epochs training for *MNIST* and *Fashion-MNIST* respectively. Toast plot (**a**) shows the *LeNet 4x300*, (**b**) *G(n,p) 1194*, (**c**) *C. elegans*^26^ and (**d**) the human *connectome*^27^. In all four networks broad yellow regions indicate very few changes in the edges between different initalization settings. Widespread black or dark grey regions in the lower left toasts exhibit convergence in its active weights. This effect is much more conserved in the recurrent networks, but also the feed-forward networks show this effect blockwise within one initalization method. The minimum distances for a 95% significance are based on t-test. R—reference, g—Glorot, L—lightning , D—dense diversity, S—sparse diversity initialization; c—connectome, C—*C. elegans*; I—iterative pruning, B—bio pruning; W—winning ticket, i—structure implantation.

A final argument underlining the importance of structure is illustrated in Fig. 4 (for a detailed description of the ‘toast plots’ and its analysis see “Methods”). It turned out that for each initialization the resulting structure was always analogous to the structure with which it was initialized, independent of the experimental settings.

This phenomenon was particularly conserved in recurrent networks. Here, only a minimum of edge changes could be detected in the entirety of the connections. Also the Spearman R coefficient, which is depicted in the lower left toast and illustrates the change of weights, shows that these structures also were almost completely similar in their active weights. The lack of correlation for not pruned and implanted information (winning ticket and structure implantation) results from the analysis of the primary (part of the structure) and secondary (not part of the structure) connections, since the secondary part, which is random, is not deactivated by pruning. It thus can be concluded that the weights will follow through the implanted structure.

To give a first summary, presenting the arguments for one side of the dilemma, it can be stated that it is possible to initialize a network with a pre-learned structure, missing the information of weights, with an equal accuracy and an improved learning behavior. In addition, the edge and weight comparison of unmodified, pruned and implanted models repeatedly reveal the fact that the structure holds the information about the weights itself. As it could be seen by the transformation of a biological network into an artificial network learning successfully, biological network structure is conserved and seems to play an important role. We were thus able to show that the importance of structure is not only a phenomenon in naturally occurring, but also in artificial information processing structures.

### Structure is not exclusive

Reconsidering the figures presented thus far, it must be noted, however, that these also contain arguments for the other side of the paradox. It could be seen that the structure—although being of considerable importance—is not exclusive. For instance, it is evident from Fig. 4 that the same structure was able to solve both problems, the *MNIST* and the more complex *Fashion-MNIST*. This is indicated by the extensive yellow areas in the upper right toast, which for the most part show only minimal changes in the connections. This effect of so-called *transfer-learning* has been observed in recurrent networks as well as in the feed-forward networks. It is remarkable that not only the structure was left unchanged during learning of an alternative problem, but that also the weights were nearly identical (indicated through dark black areas in the lower left toast). Therefore, a more or less randomly initialized network could solve two different problems with nearly the same structure and weights. The same applies to *C. elegans*. As it was mentioned in the previous section, the worm was not made to solve this synthetic problems, neither its sensory neurons were made to read image information, nor its motor neurons to output numbers. Nevertheless, also in its pruned configuration, it was able to solve this task.

Also in Fig. 3, some arguments underline the non-exclusivity of structures. Thus, if the bar graph is considered in its entirety, it shows that every network can solve the two problems. Only a minimal difference of less than 5% could be observed within one calculation experiment between the networks. Especially remarkable in this context were the random *G*(*n*, *p*) graphs. Although these had by definition random connections and thus randomized structures, they did not perform worse in solving the problems. On the other hand, even the more sophisticated networks showed very small improvements or even a worse behavior in their accuracy. This lead to the hypothesis that probably any structure can be used to solve any problem, as long as the network has enough capacity. Indeed, this finding is not entirely new. Honey and colleagues claimed that a variety of functional roles can be performed by ‘computational reservoirs’ with sufficient built-in complexity^1^. It should be emphasized that this theory was also discussed and supported on the biological side by various studies. For example, Prinz et al.^37^ simulated the pyloric rhythm of the crustacean stomatogastric nervous system and found that this even tightly regulated network can result from a wide range of different underlying mechanisms and parameters. Biological networks with structural^38^ and functional^39^ variability can perform the same functions. In addition, there are findings that neurons themselves rather than their connections are tuned in the learning process. Nonsynaptic changes of membrane components, such as a modulation of voltage-dependent membrane conductances, which results in a change in excitability, is produced by learning^40^.

Comparing structure and their weights alone misses the point of isomorphisms. A graph can be identically connected but on a different node setting. This may appear during the analyses of edge changes and the correlation of weights as a different network. The upper right toasts in Fig. 6 show the results of a simple isomorphism test. If two graphs are isomorph, they have an identical degree sequences over all nodes. A match indicates that both graphs could be isomorph, a mismatch denotes that the graphs were not isomorph (further information on the isomorphism test can be found in “Methods”). Figure 6 shows that in most cases the variants were not isomorph. Only few pairs displayed the possibility to be identical.

### The influence of the weights and the neuron offset value

Considering the results presented thus far showing the two sides of the paradox, the question of what influence the weights have on the results remains unresolved. In fact, in all of the networks investigated, the best possible accuracy between *winning ticket* and *structure implantation* was found to be very similar. Both problems, *MNIST*^41^ and *Fashion-MNIST*^42^, were solved with identical accuracy for each data set, which disclosed the relevance of the structure even without the weights. This was additionally supported by Fig. 4, showing that during learning only minimal changes were applied to the weights.

**Figure 5:**
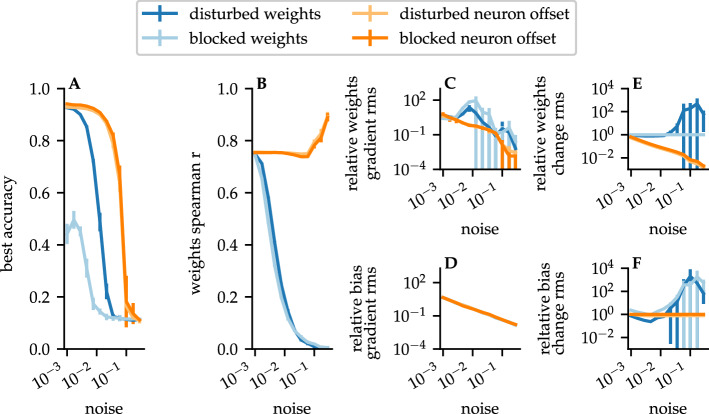
Experiments with disturbed learning for a uniform *1194*. Shown are results for blocked or disturbed weights or neuron offset. Error bars show standard deviation over ten independent random iterations. (**A**) Best accuracy in relation to the added noise. (**B**) Spearman R between the trained and untrained weights in relation to the added noise, to show the similarity of the weights. The effective value of the weight (**C**) and neuron offset (**D**) gradients relative to the noise demonstrates the relation of gradients to the added noise, to show the effective change per step. The relative, effective weights (**E**) and neuron offset (**F**) change shows the development of the RMS value related to the noise, to illustrate the change over the complete training.

Indeed, a quite familiar observation was drawn from Gaier and Ha^25^ as they figured out the importance of the architecture in contrast to the previously established interest in the weights. To put this influence of the weights to the test, we performed experiments with either disturbed or blocked weights and neuron offset values, respectively. A disturbance of the value means that a normally distributed noise was added before each learning step. In blocked runs the disturbed values were not trainable (further information can be observed in the “Methods” section). Figure 5 shows the results of this experiment. Despite adding noise to the weights, the best accuracy decreased only slightly up to a level of approximately $10^{-2}$ (dark blue). If the added noise was greater than $10^{-2}$, the accuracy dropped rapidly. As can be seen in Fig. 5E,F on this scale a range of instability is recognizable. The solver is provoked to make changes that are even disproportionately stronger in the range over $10^{-2}$ than the actual change caused by the noise.

For the blocked runs, the behaviour is similar, except that the best accuracy is only at around 0.5. Here the role of the bias is also indicated. Although weight changes can no longer be made, the solver is still able to change the neuron offset value and thereby reaches an accuracy of 0.5. The role of bias should thus not be underestimated.

The neuron offset value was even less sensitive to the disturbances. In both cases, disturbed and blocked bias values, the best accuracy was stable up to a strength of $10^{-2}$. If the results from Fig. 5E,F are also taken together, it can be seen that the neuron offset values changed by the noise are hardly changed by the solver. Changes in the bias can be easily compensated by minimal changes in the more powerful weights, as can be seen in Fig. 5B. Until the system gets unstable, the weights remain constant in the relation to the starting point. Above the stability point the weight change gets smaller, because the solver is unable to find the correct countermeasure to compensate the disturbance. The initial configuration in the weights, which follow directly from the structure, thus can not be disturbed severely without the system becoming unstable, as can derived from Fig. 5A,B. With the described conclusion that the weights follow from the structure, it must therefore be noted that this solution has only a small domain.

**Figure 6:**
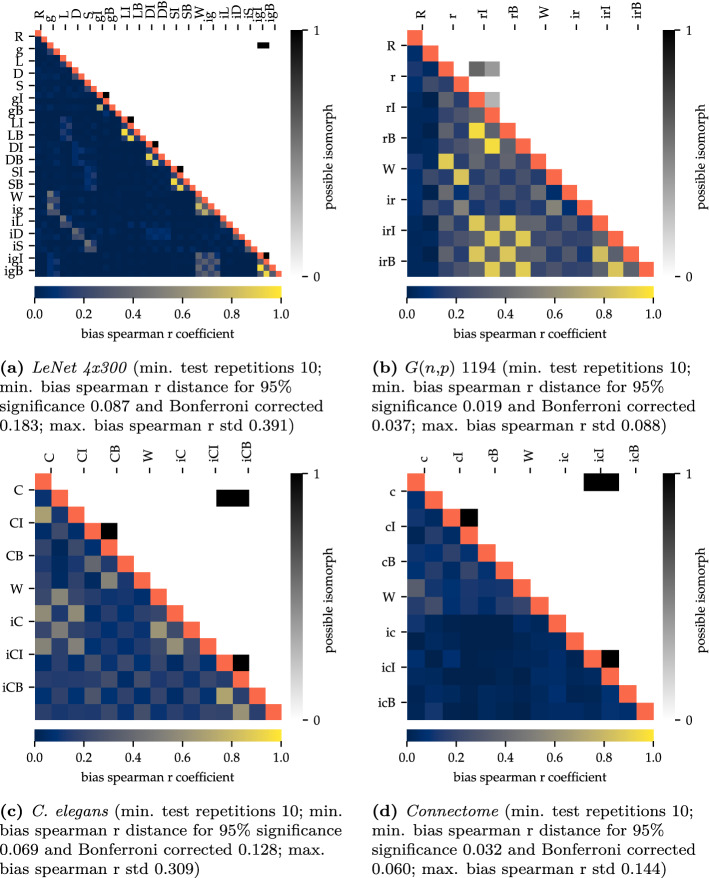
Toast plot type 2: Bias, means neuron offset value, change and verification of a possible existing isomorphism during learning and between different initial states. Identical tests marked red. Every mark consists of the configuration after 30 epochs training for *MNIST* and *Fashion-MNIST* respectively. Toast plot (**a**) shows the *LeNet 4x300*, (**b**) *G(n,p) 1194*, (**c**) *C. elegans*^26^ and (**d**) the human *connectome*^27^. The majority of dark blue coloring in the lower left toast clearly show the comprehensive change in the bias value that have taken place in each artificial neuron. The upper right toasts show the results of the isomorphism test, which states the possibility that a graph could be isomorph. In most cases the calculation excluded this. The minimum distances for a 95% significance are based on t-test. The minimum distance for a 95% significance for the possible isomorphism is 0.9 based on binomial test R—reference, g—Glorot, L—lightning , D—dense diversity, S—sparse diversity initialization; c—connectome, C—*C. elegans*; I—iterative pruning, B—bio pruning; W—winning ticket, i—structure implantation.

This may be also the reason why only very few changes in the weights were detected in the learning process. However, if it turns out that neither the structure nor the resulting weights undergo major changes in the learning process, the question arises how artificial neural networks are able to learn at all. In principle, there are two possibilities. First, it is possible that even minimal changes could have enormous effects on the entire system, i.e. it could be a chaotic system. The other, perhaps more likely explanation could be an adjustment of the neuron offset value. The bottom left toasts of Fig. 6, in which the Spearman R coefficient of the neuron offset values are displayed, providing an exemplary answer to this question for four of our analysed networks. Independent of the network, the Spearman R correlation coefficient showed mostly very low values (recognizable by an almost completely dark blue coloration), indicating the dissimilarity between individual neuron offset values. This clearly demonstrated the change in those values caused by the learning process. Thus, it could be stated that adjustments were done by the solver not as commonly assumed largely through weight or structural changes, but through changes in the neuron offset value of each artificial neuron.

This behavior could be interpreted as an increasing or decreasing sensitivity of every individual neuron to its input values. This behaviour suggested that a change in the neuron offset value was sufficient to adjust the network effectively.

In a biological context, a possible analogy for this phenomenon can also be identified. A modulation through an intracellular storage of calcium can result in a short-term signal amplification as well as an adjustment of neuronal excitability^43^. Although the behaviour is similar, it is doubtful whether gradient-based learning as a mechanism can be compared with learning performed by true nervous systems. Therefore, the obviously similar behaviour might have a completely different cause.

Nonetheless, the further development of artificial neural networks can make use of known functions from nature in order to extend their functionality and effectiveness in a sustainable way. From a biological point of view, results, obtained through ongoing technical simulation experiments, may also contribute to the understanding of the learning process and the structural organisation of the brain.

## Discussion

In this paper we wanted to understand the relevance of the structure for information processing. Therefore, we analysed the behavior of artificial and biological-based networks based on technical neurons and their training methods. We were able to show that the structural dilemma, which is often resumed in biology, can also be found in ANNs. We figured out that an initial structure is indeed sufficient to improve learning accuracy, with minimal importance on the weights. Nevertheless, it seems to be of less relevance how this structure is organized. Contrary to expectations, a network based on biological structural principles does not improve learning success compared to randomly initialized structures. This raises the question why biological networks show precisely these structures. Based on our findings, it is questionable that these structures are made specifically for information processing. But, this follows the question why these structures are created that way and why deviations show pathogenic patterns. To answer these questions, further experiments will be carried out based on the parallels between artificial and biological-based neural networks presented in this paper. For this it is essential to include further information levels of the biological system. Further research is therefore aimed to model the technical neurons in a more biological way based on neural coding^44,45^, or to include electrical synapses.

## Methods

### Biological network data

For a multi-level analysis and comparison of biological networks we used two different datasets. First, the wiring diagram of the male *C. elegans* (updated dataset: https://wormwiring.org/series/ published in^26^), representing the micro-connectome level, and second, functional *connectome* data of healthy patients with varying age, gender and ethnical background, representing the macro-connectome level^36^, (directly accessible via: http://umcd.humanconnectomeproject.org/umcd/default/browse_studies)^27^. Both data sets are freely accessible online.

The single data set of the male *C. elegans* is composed of 369 neurons, 3888 connections through chemical synapses, 134 muscle connectors and 1018 muscle connections. The analysed adjacency matrix is bidirectional and weighted. More details about this dataset can be obtained from^26^.

984 data sets were included in the analysis of the macro-connectome, as 17 of the provided sets were not complete. In particular, the data records of “Beijing_Zang_newid182” with the sequential number up to 198 were affected, when we downloaded the data in August 2019. We processed the provided adjacency matrix with a threshold to obtain a mean degree of around 22. This results in a number of edges of 3964. The connectome graph was undirected but weighted. We ignored the weights, because we were only interested in the structure. The undirected graph was transformed to a directed one by randomly choosing the direction and type equally for all edges.

### Artificial neural network data

In order to make well-founded statements about the behaviour of ANNs, we used both feed-forward as well as recurrent networks. As feed-forward networks, we used the established structures of the *LeNet 300-100* and the *LeNet 4x300*^46^. For the class of recurrent networks, we used random *G*(*n*, *p*) graphs with either 1194 nodes corresponding to the *LeNet 300-100* architecture and a comparable mean degree, or 177 nodes analogous to the *connectomes* and also a corresponding mean degree. These graphs were calculated additionally with a directed small-world architecture.

The adjacency matrices of the human connectome were preprocessed first, as it was described in the previous section. For *C. elegans*, we used the naturally occurring network, provided from the original dataset, and a randomly connected version as *G*(*n*, *p*) with an equal number of nodes. A speciality of the *C. elegans* model are the separated layers for neurons (including sensor neurons) and muscle attachment points. The *G*(*n*, *p*) reference holded the muscle layer and connects the neuron layer randomly. The dataset of the *C. elegans* holded no information about excitatory and inhibitory neurons. Based on a calculated, theoretical optimal percentage of inhibitory synapses of 30%^47^ and a measured value of typical 20–30%^48^ in mammalian brains we assumed this percentage also for *C. elegans*. We trained 10 different datasets with a random value between 20 and 30% of inhibitory neurons. We saved this model, but did not perform a Monte Carlo simulation to avoid over-interpreting of the distribution of the inhibiting neurons.

### Training process

#### Initialization

In order to test the effect of different conditions at the beginning of learning, the nets were initialized differently. The methods included the randomized initialization *glorot*^49^, *lightning*^28^, *winning ticket*^17^ and two new implemented methods called *structure implantation* and *diversity*. In contrast to *winning ticket*, in which a pretrained structure including its weights has been implanted the *structure implantation* only implanted the structure with the categories exciting, inhibiting and no connections but without any information about the weights. This was done by using a threshold to decide whether or not an edge in the graph existed. This threshold was adjusted dynamically. The *n* strongest connections (positive and negative) were used to keep the average connectivity *c* constant. The existing edges were divided in positive and negative weights, corresponding to an exciting or inhibiting connection. Non-existing edges were 0, existing ones were set to the same value with the sign corresponding to the connection type. Low noise of a tenth of the connection value was added on top of the resulting adjacency or bipartite matrices.

The method of the *diversity* initialization should represent first degrees of kinship with biology. The amount of links showed a distribution in a specific range and not a constant value. The *diversity* initialization generated a set of neurons with a randomly chosen number of edges to random other neurons. For this paper this type of initialization was again divided into two subgroups:

*Dense diversity*: very dense connected system defined by the in-going degree $d_\text {in}$.
  1 $$\begin{aligned} d_\text {in} = {\left\{ \begin{array}{ll} \left[ {\mathcal {N}}\left( 100, 10\right) \right] &{} \quad \text {for } 35\% \text { off all existing Neurons}\\ \left[ {\mathcal {N}}\left( 50, 5\right) \right] &{} \quad \text {otherwise} \end{array}\right. } \end{aligned}$$
*Sparse diversity*: sparse connected system defined by the in-going degree $d_\text {in}$.
  2 $$\begin{aligned} d_\text {in} = {\left\{ \begin{array}{ll} \left[ {\mathcal {N}}\left( 8, 2\right) \right] &{} \quad \text {for } 80\% \text { off all existing Neurons}\\ \left[ {\mathcal {N}}\left( 25, 5\right) \right] &{} \quad \text {otherwise} \end{array}\right. } \end{aligned}$$

#### Datasets

The artificial neural networks were trained on either the *MNIST*^41^, *Fashion-MNIST*^42^ or the *CIFAR-10*^50^ (For *CIFAR-10* the results are available in the Supplementary Materials) database. Recurrent networks iterated over a specific number of calculation steps. All recurrent networks were calculated for 25 steps and the input information was constantly set. The values of the datasets were normalized to the range of $\left[ 0, 1\right]$ as a preprocessing step.

#### Architectures

As already described in the beginning of this chapter, we used different ANNs for our calculations.

- *LeNet 300-100:* 784 input neurons followed by a dense layer with 300 neurons, a dense layer with 100 neurons and 10 output layer neurons containing the output information; only the output layer had a softmax activation function, whereas the others were activated through relu.
- *LeNet 4x300*: 784 input neurons followed by 4 dense layers with 300 neurons each and 10 output neurons; only the output layer had a softmax activation function, whereas the others were activated through relu.
- *Recurrent 1194*: 1194 neurons were activated through tanh; 784 of the 1194 were defined as input neurons, to generate the output 10 neurons were calculated with softmax in an additional output layer.
- *Recurrent 117*: The 784 input information of the datasets were encoded with an random encoder with a distribution of ${\mathcal {U}}(-0.05, 0.05)$, the same encoder was used for all experiments, all neurons were input neurons, to generate the output 10 neurons were calculated with softmax in an additional output layer.
- *C. elegans model*: The 784 input information of the datasets were encoded with an random encoder with a distribution of ${\mathcal {U}}(-0.05, 0.05)$ to 136 sensory neurons, which were included in the recurrent layer with 369 tanh activated neurons, which were mapped to the 134 tanh activated muscle attachment points, those were decoded to 10 output values which were calculated with softmax in an additional output layer. In general, it needs to be added that the given connectivity matrix values were, due to the absence of inhibiting connections and a to wide numerical range not suitable to use, which made the definition of other values necessary.
- *C. elegans LeNet model*: The en- and decoding is the same as in *C. elegans*. The minimal distances of every node to an output was calculated and based as mapping for the feed forward layers. The encoded inputs were also added based on the minimal distance to an output mapping. Thus, this model has several inputs on all four layers. The exact configuration is shown in Table 7. 22 nodes are not added to the model, because they are not connected in the *C. elegans* micro-connectome.

**Table 7:** *C. elegans* LeNet architecture.

Inputs	Hidden nodes	Outputs	Activation
3	0	0	–
23	12	0	relu
71	64	0	relu
35	139	0	relu
0	0	134	softmax

#### Pruning

We used two different pruning techniques: *iterative pruning* and the newly introduced *bio pruning*. During the *iterative pruning* the graph was analysed after each epoch and the pruning mask was updated to the new graph. In opposite to that the *bio pruning* was aimed to take up another idea from biology, the random based creating and destroying of connections. The solver was allowed to build up maximum $\mathrm {Pois}(20)$ new connections, delete maximum $\mathrm {Pois}(20)$ weak connections and could break 0 strong connections after each epoch. Within the number of connections, resulting from the average connectivity *c*, strong connections can be formed. Beyond of this, weak connections can be built up and destroyed. The average connectivity may therefore variate for the *bio pruning* method.

#### Training

The models were trained with the solver settings given in Table 8. Categorical cross entropy was used as loss value and accuracy as metric. All models were trained for 30 epochs with shuffled batches of the size 128 for the feed forward and 256 for the recurrent networks.

**Table 8:** Solver parameter for different architectures.

Architecture	Solver	Learning rate	Momentum
*LeNet 300-100*	SGD	0.050	0.00
*LeNet 4x300*	SGD	0.050	0.00
*G*(*n*, *p*) & *small world* 1194	SGD	0.030	0.01
*irnn* & *uniform* 1194	SGD	0.050	0.01
*G*(*n*, *p*) & *small world* 177	SGD	0.050	0.00
*irnn* & *uniform* 177	SGD	0.050	0.01
*C. elegans* model	SGD	0.075	0.01

All variants that we trained have learned to solve the problems. We only figured out two exceptions, which were the pruned *glorot* initialized feed forward networks with a mean degree less than 15. This can be observed in Table 9. The table shows the behavior of the *glorot* initialization based on the mean degree for the *LeNet 300-100*.

A weakly connected component is the amount of nodes that have paths between each other independent of the edge directions. If the fraction of the largest weakly connected component $S = 34.3\%$, only a third of the neurons were connected to each other in maximum.

**Table 9:** Comparison of best validation accuracy and fraction of the largest weakly connected component *S* for *glorot* and *lightning* initialization for LeNet 300-100 with different mean degree *c*.

*c*	Pruning value (%)	Experiment	Best accuracy		*S*	
5		*Glorot*	$98.0\%$	$\pm 0.059\%$	$34.3\%$	$\pm 0\%$
5		*Lightning*	$98.0\%$	$\pm 0.086\%$	$99.6\%$	$\pm 0.119\%$
5	2.2	Pruned *glorot*	$11.3\%$	$\pm 0\%$	$34.3\%$	$\pm 0\%$
5	2.2	Pruned *lightning*	$93.3\%$	$\pm 0.318\%$	$99.6\%$	$\pm 0.119\%$
10		*Glorot*	$98.0\%$	$\pm 0.067\%$	$34.3\%$	$\pm 0\%$
10		*Lightning*	$98.1\%$	$\pm 0.068\%$	$100.0\%$	$\pm 0\%$
10	4.5	Pruned *glorot*	$11.3\%$	$\pm 0\%$	$34.3\%$	$\pm 0\%$
10	4.5	Pruned *lightning*	$95.1\%$	$\pm 0.239\%$	$100.0\%$	$\pm 0\%$
15		*Glorot*	$97.9\%$	$\pm 0.056\%$	$99.9\%$	$\pm 0.097\%$
15		*Lightning*	$98.1\%$	$\pm 0.092\%$	$100.0\%$	$\pm 0\%$
15	6.7	Pruned *glorot*	$95.3\%$	$\pm 0.187\%$	$99.9\%$	$\pm 0.097\%$
15	6.7	Pruned *lightning*	$95.9\%$	$\pm 0.138\%$	$100.0\%$	$\pm 0\%$
21.9		*Glorot*	$98.0\%$	$\pm 0.064\%$	$100.0\%$	$\pm 0\%$
21.9		*Lightning*	$98.0\%$	$\pm 0.056\%$	$100.0\%$	$\pm 0\%$
21.9	9.8	Pruned *glorot*	$96.1\%$	$\pm 0.136\%$	$100.0\%$	$\pm 0\%$
21.9	9.8	Pruned *lightning*	$96.5\%$	$\pm 0.180\%$	$100.0\%$	$\pm 0\%$

±-values show standard deviation over ten independent random iterations.

A reason for the learning deficiency of the pruned *glorot* variant could be the fact that the largest weakly connected component was to small. The other feed forward initialization (*lightning*, *dense* and *sparse diversity*) did not show this behaviour. The values of the diversity initialization were similar to *lightning*.

### Disturbed and blocked training

Gradient based learning tries to minimize the loss function *l* by adjusting the trainable parameters. This is achieved by following the gradient $\frac{\partial l}{\partial x_i}$. If the learning parameters are set well, the optimizer climbs down to a local minimum by every step. Based on the start parameter the local minimums can differ. By adding noise to the trainable parameters the optimizer is forced to react on this disturbance. Related to the strength of the noise a solution is possible by compensating the distribution or climbing down an other local minimum.

In our experiments the normal distributed noise $\epsilon \sim {\mathcal {N}}(0, \sigma )$ with a mean of 0 and a standard deviation of $\sigma$ is added to the values $\chi$ before the optimization in every batch. Weights and neuron offset are treated separately and were not disturbed together. Thus, the optimizer has to deal with the disturbed weights or altered neuron offsets. Additionally, to show the compensating of the optimizer, in blocked runs the disturbed values were not trainable. The optimizer has to compensate the disturbance only by adjusting the unblocked values. The unblocked values were not disturbed. As architecture a pruned *recurrent 1194* with an *uniform* initialisation were used. Two metrics were used:

*Relative gradient rms*: This metric shows the strength of the gradient related to the added distrubtion.
  3 $$\begin{aligned} \gamma = \frac{\sqrt{\frac{1}{n}\sum _{i=1} ^n \frac{\partial l}{\partial \chi _i}}}{\sigma } \end{aligned}$$
*Relative change rms*: This metric shows the effective change of the values to the initial state by the gradient related to the effective change of the noise over *N* batches.
  4 $$\begin{aligned} \delta = \frac{\sqrt{\frac{1}{n}\sum _{i=1} ^n \Delta \chi _i}}{\sqrt{N} \sigma } \end{aligned}$$

### Possible graphs and isomorphism

The difficulty in calculating an isomorphism is demonstrated in Fig. 6d. Per definition, the connectome-based recurrent networks can not have isomorph graphs, because these networks have no free nodes that are not connected to an input or output. The isomorphism test checks only the possibility of an isomorphism in general. It cannot test against the specific conditions of fixed input and output nodes. So the algorithm tends to show a higher possibility that an isomprophism exists than actually would be the case. A discussion of the absolute changes in the graphs requires a reference in order to be able to estimate how large a change actually is. For this purpose different theoretical values of the graphs were calculated. The number of possible edges

5 $$\begin{aligned} M = \sum _{l = 0}^{L} n^{l}_\text {ingoing} \cdot n^{l}_\text {outgoing} \end{aligned}$$

with *L* number of layers and $n^{l}_\text {ingoing}$ or $n^{l}_\text {outgoing}$ number of nodes in- and outgoing of layer *l* defines the amount of all possible connections. The number of all possinble unique graphs

6 $$\begin{aligned} P\left( M, m \right) = \frac{M!}{m!} \end{aligned}$$

with *M* as number of all possible edges and *m* as number of existing edges, shows how many unique forms the graph could have. For this we assume that free nodes, which are not connected to an input or output, can permuted in their position. The last paramater is the number of possible isomorph graphs

7 $$\begin{aligned} I = \prod _{l = 0}^{L} f^{l}! \end{aligned}$$

with $f^{l}$ as number of free nodes in layer *l*.

Table 10 (calculated with https://www.wolframalpha.com/) showed the permutation and isomorphisms values for the different architectures, which were used in this paper. The selected architectures covered a wide range and differ in their basic properties. For example, the *recurrent 177* architecture had relative few possible edges and unique graphs and no free nodes. In opposite, the *recurrent 1194* architecture had the most possible edges and unique graphs but not as much isomorphisms as the *LeNet 4x300* architecture.

**Table 10:** Amount of possible edges, unique and isomorphic graphs for the different network architectures.

Architecture	Possible edges	Possible graphs		Isomorphisms
*LeNet 300-100*	266, 200	$1.71\cdot 10^{1,223,900}$	For $c = 22$	$2.86 \cdot 10^{772}$
*LeNet 4x300*	508, 200	$6.02 \cdot 10^{2,494,508}$	For $c = 22$	$8.77 \cdot 10^{2,457}$
Recurrent 1194	1, 425, 636	$6.90 \cdot 10^{8,049,549}$	For $c = 22$	$6.40 \cdot 10^{868}$
Recurrent 177	31, 329	$1.38 \cdot 10^{17,708}$	For $c = 22.4$	No free nodes
*C. elegans* model	185, 607	$4.21 \cdot 10^{881,304}$	For $c = 9.75$	$1.46 \cdot 10^{659}$

### Analytical methods

#### Toast plots

**Figure 7:**
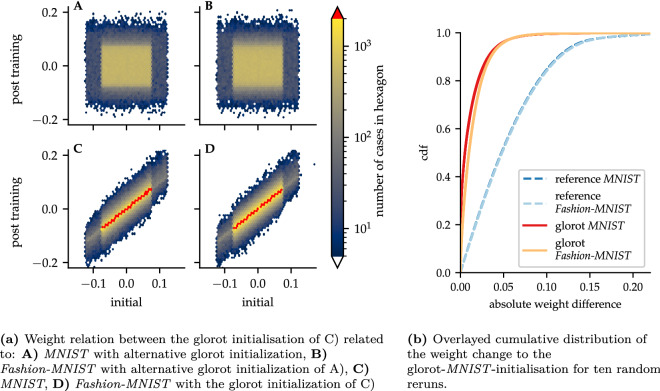
Weight relation of a *LeNet 300-100* architecture between initialisation and post trained state.

To observe the changes in the trained model with simple metrics, we focus on the change of the weights during the learning. Figure 7a shows the distribution of the weight dependencies between different states of the model. By comparing two independent initialized states the weights show no logic and distributed equally corresponding to the different layers. The comparison of the initial to the corresponding trained state shows the complete opposite. The post training weights depend directly on the initialisation. Figure 7b show the cumulative distribution function of the weight change between the different states of Fig. 7a. It demonstrates the gap between randomized and depending trained weights.

**Figure 8:**
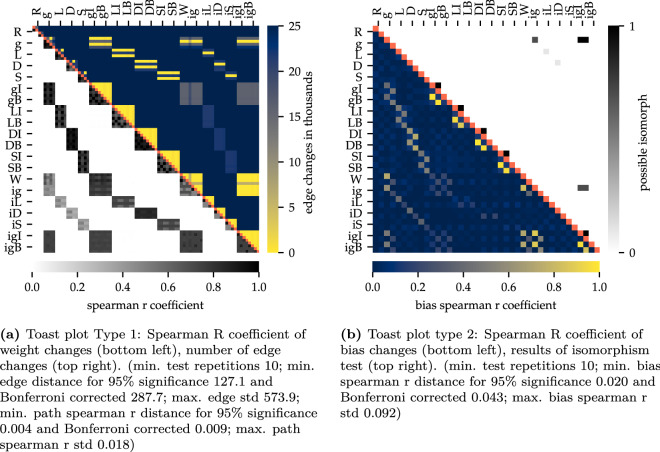
Demonstration of the two types of toast plots. Exemplary for the feed forward network *LeNet 300-100*. The minimum distances for a 95% significance are based on t-test. The minimum distance for a 95% significance for the possible isomorphism is 0.9 based on binomial test.

For an optimized presentation of the extensive analytical results we developed our so-called toast plots. Figure 8 shows the two types we used in this paper. They offer the advantage that several analyses as well as runs can be recorded and compared at a glance. One plot is built out of two toasts. In particularly, type 1 (see Fig. 8a) displays the Spearman R coefficient of the edge changes, meaning how similar the weights of the edges are compared to other runs. The second toast of that plot shows the number of odd edges compared to other runs. Type 2 (see Fig. 8b) shows the Spearman R coefficient of the bias values and the results of an isomorphism test (see section ‘Possible graphs and isomorphism’ for further details).

Each run is marked with a one to three letter abbreviation at the side of the graph. Each of these include four different subvariants, entailing the data set they were trained with (see more details in training section) and if it is the initial or the trained status. The abbreviations given in the legends describe how the net was initialized and pruned. In detail these are: R—reference, g—Glorot, L—lightning , D—dense diversity, S—sparse diversity initialization; c—connectome, C—*C. elegans*; I—iterative pruning, B—bio pruning; W—winning ticket, i—structure implantation. A detailed explanation of the abbreviations and setups are given in the training process section.

## Supplementary Information

Supplementary Information.

## Data Availability

The artificial datasets generated during and analysed during the current study are available from the corresponding author on reasonable request. The biological datasets from *C. elegans* is freely available and can be downloaded from: https://wormwiring.org/series/. The human connectome dataset is freely available and can be downloaded from: http://umcd.humanconnectomeproject.org/umcd/default/browse_studies.
