# Supplementary material for: The structure dilemma in biological and artificial neural networks
Source: Sci Rep. 2021 Mar 10;11:5621. doi: 10.1038/s41598-021-84813-6 (PMC7970964; doi:10.1038/s41598-021-84813-6)
Supplement: Supplementary file 1 — Supplementary Information. [file 41598_2021_84813_MOESM1_ESM.pdf]

The structure dilemma in biological and artificial neural networks

Thomas Pircher[1,*,+], Bianca Pircher[2,+], Eberhard Schlücker[1], and Andreas Feigenspan[2]

[1]Institute of Process Machinery and Systems Engineering, Friedrich-Alexander University Erlangen-Nuremberg, Cauerstraße 4, 91058 Erlangen, Germany
[2]Department Biology, Animal Physiology, Friedrich-Alexander University Erlangen-Nuremberg, Staudtstraße 5, 91058 Erlangen, Germany
[*]pi@ipat.fau.de
[+]these authors contributed equally to this work

Supplementary Information

Additional calculation results

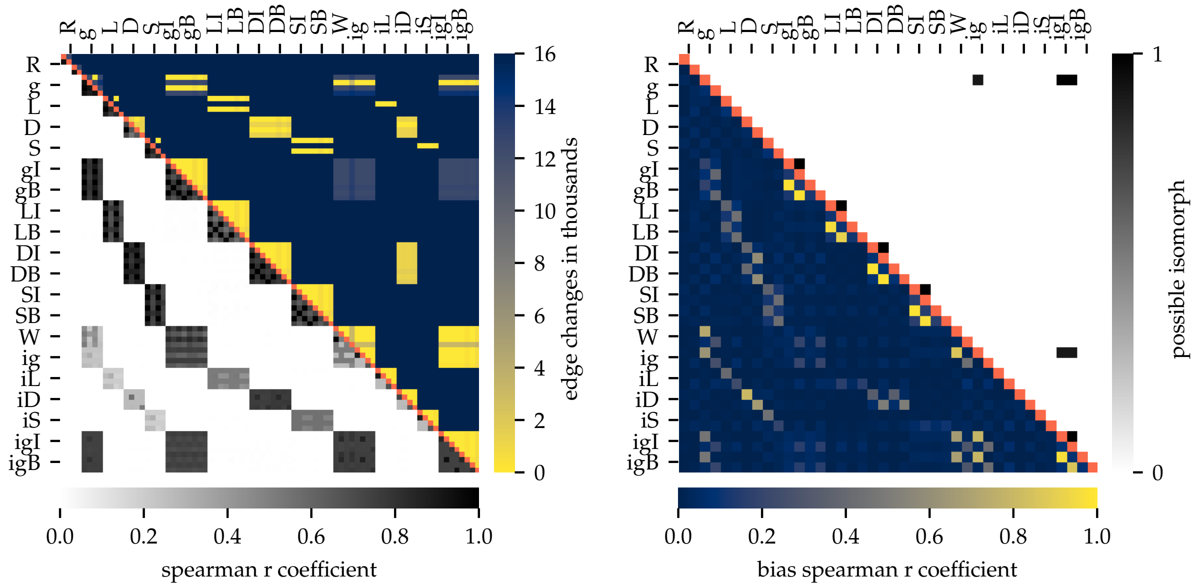

(a) Toast plot Type 1: Spearman R coefficient of weight changes (bottom left), number of edge changes (top right).

(b) Toast plot type 2: Spearman R coefficient of bias changes (bottom left), results of isomorphism test (top right).

Figure S1. Feed forward network *LeNet 300-100* mean degree 22

Table S1. Comparison of best validation accuracy and fraction of the largest weakly connected component *S* for *LeNet 300-100* mean degree 22 dataset. ±-values show standard deviation over ten independent random iterations.

	MNIST				*Fashion-MNIST*			
**ID**	**Best accuracy**		*S*		**Best accuracy**		*S*	
R	97.98%	±0.04%	80.55%	±0.29%	88.77%	±0.12%	96.86%	±0.21%
g	98.02%	±0.06%	80.48%	±0.39%	88.74%	±0.18%	96.87%	±0.34%
L	98.02%	±0.06%	94.82%	±0.24%	89.05%	±0.18%	99.09%	±0.32%
D	97.96%	±0.10%	100.00%	±0.00%	88.82%	±0.15%	100.00%	±0.00%
S	97.94%	±0.13%	99.36%	±0.21%	88.76%	±0.16%	99.88%	±0.08%
gI	96.08%	±0.14%	100.00%	±0.00%	85.98%	±0.11%	100.00%	±0.00%
gB	96.09%	±0.13%	100.00%	±0.00%	85.98%	±0.14%	100.00%	±0.00%
LI	96.52%	±0.18%	100.00%	±0.00%	86.58%	±0.19%	100.00%	±0.00%
LB	96.54%	±0.17%	100.00%	±0.00%	86.64%	±0.18%	100.00%	±0.00%
DI	96.20%	±0.19%	100.00%	±0.00%	86.16%	±0.14%	100.00%	±0.00%
DB	96.21%	±0.12%	100.00%	±0.00%	86.08%	±0.14%	100.00%	±0.00%
SI	95.78%	±0.11%	100.00%	±0.00%	85.72%	±0.18%	100.00%	±0.00%
SB	95.76%	±0.07%	100.00%	±0.00%	85.71%	±0.22%	100.00%	±0.00%
iW	98.22%	±0.04%	80.48%	±0.39%	89.00%	±0.14%	88.33%	±0.35%
ig	98.05%	±0.03%	80.48%	±0.39%	88.73%	±0.13%	83.02%	±0.35%
iL	98.06%	±0.05%	94.82%	±0.24%	88.74%	±0.24%	95.39%	±0.33%
iD	98.05%	±0.08%	100.00%	±0.00%	88.85%	±0.18%	100.00%	±0.00%
iS	98.04%	±0.07%	99.36%	±0.21%	88.61%	±0.18%	99.45%	±0.19%
igI	97.83%	±0.06%	80.48%	±0.39%	84.62%	±0.20%	80.48%	±0.39%
igB	97.87%	±0.05%	83.98%	±0.54%	84.79%	±0.23%	89.92%	±0.68%

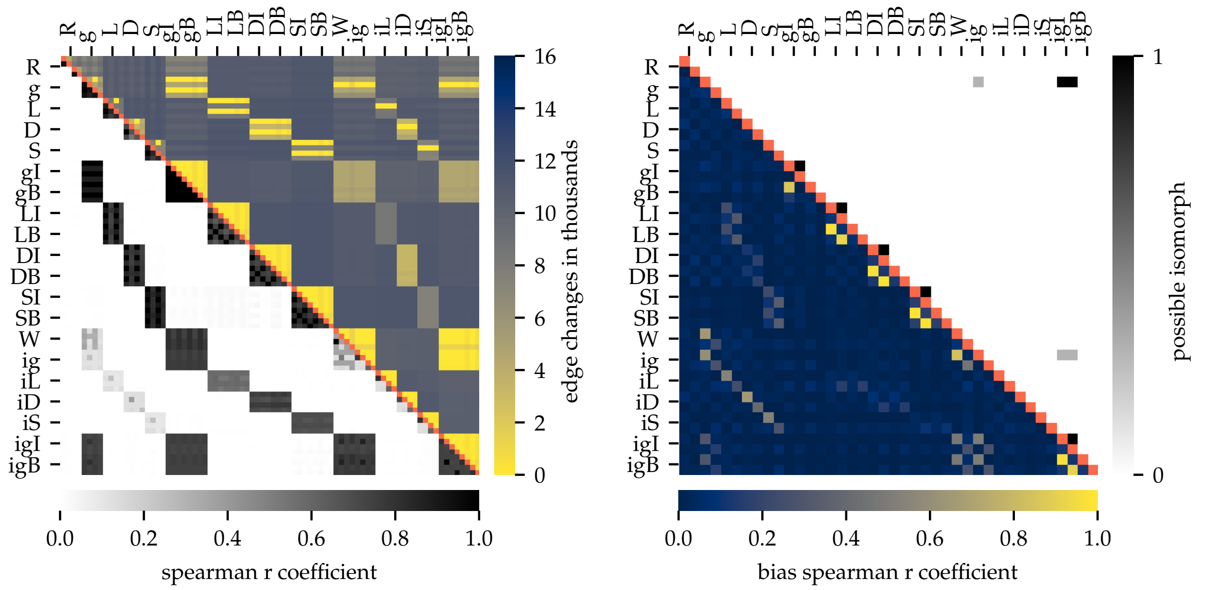

(a) Toast plot Type 1: Spearman R coefficient of weight changes (bottom left), number of edge changes (top right).

(b) Toast plot type 2: Spearman R coefficient of bias changes (bottom left), results of isomorphism test (top right).

Figure S2. Feed forward network *LeNet 300-100* mean degree 10

Table S2. Comparison of best validation accuracy and fraction of the largest weakly connected component S for *LeNet 300-100* mean degree 10 dataset. ±-values show standard deviation over ten independent random iterations.

	MNIST				*Fashion-MNIST*			
**ID**	**Best accuracy**		S		**Best accuracy**		S	
R	97.95%	±0.06%	67.13%	±0.35%	88.69%	±0.18%	88.55%	±0.28%
g	98.00%	±0.07%	67.34%	±0.31%	88.85%	±0.21%	88.10%	±0.46%
L	98.07%	±0.07%	92.04%	±0.56%	89.12%	±0.27%	97.93%	±0.35%
D	97.93%	±0.09%	99.98%	±0.04%	88.85%	±0.20%	99.99%	±0.03%
S	97.95%	±0.06%	98.95%	±0.38%	88.75%	±0.22%	99.59%	±0.16%
gI	11.35%	±0.00%	34.34%	±0.00%	10.00%	±0.00%	34.34%	±0.00%
gB	11.35%	±0.00%	34.34%	±0.00%	10.00%	±0.00%	34.34%	±0.00%
LI	95.09%	±0.24%	100.00%	±0.00%	85.08%	±0.26%	100.00%	±0.00%
LB	95.08%	±0.23%	100.00%	±0.00%	85.09%	±0.33%	100.00%	±0.00%
DI	94.21%	±0.24%	99.99%	±0.03%	84.43%	±0.13%	99.99%	±0.03%
DB	94.19%	±0.20%	100.00%	±0.00%	84.33%	±0.10%	99.99%	±0.03%
SI	94.00%	±0.18%	100.00%	±0.00%	83.89%	±0.27%	100.00%	±0.00%
SB	93.99%	±0.15%	100.00%	±0.00%	83.88%	±0.29%	100.00%	±0.00%
iW	98.28%	±0.06%	67.34%	±0.28%	88.93%	±0.15%	80.37%	±0.73%
ig	98.23%	±0.07%	67.34%	±0.31%	89.10%	±0.20%	70.39%	±0.63%
iL	98.14%	±0.05%	92.04%	±0.56%	88.91%	±0.26%	92.89%	±0.55%
iD	98.13%	±0.07%	99.98%	±0.04%	89.02%	±0.16%	99.99%	±0.03%
iS	98.12%	±0.07%	98.95%	±0.38%	88.69%	±0.15%	99.02%	±0.38%
igI	97.43%	±0.09%	67.34%	±0.31%	80.83%	±0.41%	67.34%	±0.31%
igB	97.45%	±0.12%	75.88%	±0.95%	81.85%	±0.72%	79.74%	±1.20%

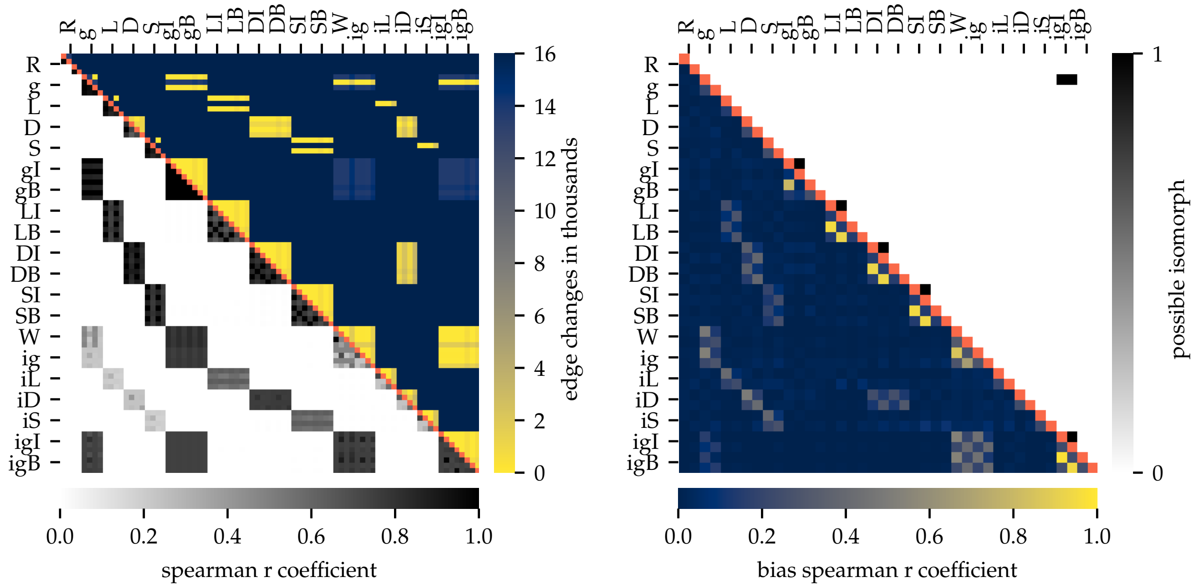

(a) Toast plot Type 1: Spearman R coefficient of weight changes (bottom left), number of edge changes (top right).

(b) Toast plot type 2: Spearman R coefficient of bias changes (bottom left), results of isomorphism test (top right).

Figure S3. Feed forward network *LeNet 4x300* mean degree 22

Table S3. Comparison of best validation accuracy and fraction of the largest weakly connected component *S* for *LeNet 4x300* mean degree 22 dataset. ±-values show standard deviation over ten independent random iterations.

	MNIST				*Fashion-MNIST*			
**ID**	**Best accuracy**		*S*		**Best accuracy**		*S*	
R	98.08%	±0.06%	82.01%	±0.38%	89.08%	±0.26%	95.71%	±0.23%
g	98.08%	±0.06%	82.01%	±0.30%	89.02%	±0.29%	95.80%	±0.21%
L	98.11%	±0.12%	99.83%	±0.16%	89.21%	±0.19%	99.16%	±0.18%
D	97.94%	±0.06%	99.20%	±0.32%	88.99%	±0.18%	99.20%	±0.32%
S	97.94%	±0.09%	99.75%	±0.14%	88.93%	±0.17%	98.48%	±0.25%
gI	11.35%	±0.00%	60.68%	±0.00%	10.00%	±0.00%	60.68%	±0.00%
gB	11.35%	±0.00%	60.68%	±0.00%	10.00%	±0.00%	60.68%	±0.00%
LI	96.81%	±0.06%	100.00%	±0.00%	86.75%	±0.16%	100.00%	±0.00%
LB	96.80%	±0.11%	100.00%	±0.00%	86.75%	±0.13%	100.00%	±0.00%
DI	96.64%	±0.13%	99.13%	±0.29%	86.57%	±0.13%	99.13%	±0.29%
DB	96.67%	±0.11%	99.13%	±0.29%	86.57%	±0.11%	99.13%	±0.29%
SI	96.36%	±0.23%	100.00%	±0.00%	86.01%	±0.10%	100.00%	±0.00%
SB	96.37%	±0.18%	100.00%	±0.00%	86.04%	±0.19%	100.00%	±0.00%
iW	98.29%	±0.06%	82.01%	±0.30%	89.30%	±0.23%	90.28%	±0.56%
ig	98.26%	±0.07%	82.01%	±0.30%	89.25%	±0.26%	89.02%	±0.32%
iL	98.17%	±0.06%	99.83%	±0.16%	89.21%	±0.24%	99.87%	±0.11%
iD	97.99%	±0.06%	99.20%	±0.32%	88.82%	±0.23%	99.20%	±0.32%
iS	98.11%	±0.06%	99.75%	±0.14%	89.00%	±0.22%	99.76%	±0.11%
igI	97.61%	±0.09%	82.01%	±0.30%	81.24%	±0.34%	82.01%	±0.30%
igB	97.63%	±0.06%	85.92%	±0.43%	81.82%	±0.64%	87.07%	±0.41%

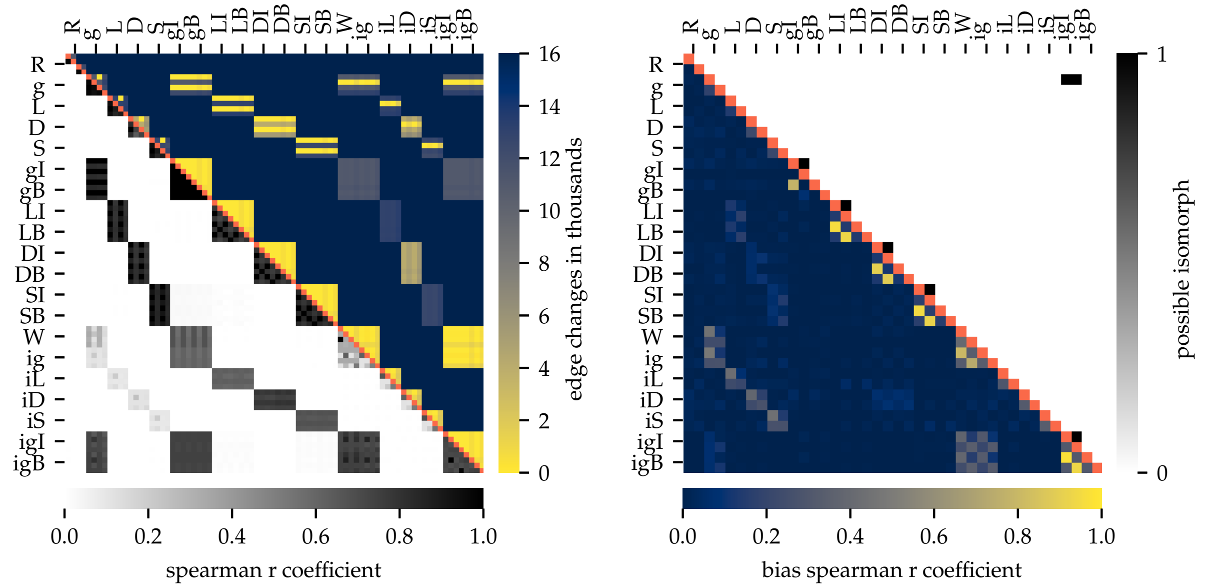

(a) Toast plot Type 1: Spearman R coefficient of weight changes (bottom left), number of edge changes (top right).

(b) Toast plot type 2: Spearman R coefficient of bias changes (bottom left), results of isomorphism test (top right).

Figure S4. Feed forward network *LeNet 4x300* mean degree 10

Table S4. Comparison of best validation accuracy and fraction of the largest weakly connected component *S* for *LeNet 4x300* mean degree 10 dataset. ±-values show standard deviation over ten independent random iterations.

	MNIST				*Fashion-MNIST*			
**ID**	**Best accuracy**		S		**Best accuracy**		S	
R	98.07%	±0.07%	77.39%	±0.29%	89.13%	±0.20%	90.35%	±0.28%
g	98.08%	±0.13%	77.38%	±0.50%	89.25%	±0.19%	90.11%	±0.36%
L	98.18%	±0.06%	94.68%	±0.34%	89.34%	±0.18%	97.72%	±0.28%
D	97.88%	±0.08%	98.38%	±0.42%	88.96%	±0.12%	98.20%	±0.35%
S	97.92%	±0.09%	96.61%	±0.42%	89.00%	±0.19%	96.46%	±0.13%
gI	11.35%	±0.00%	60.68%	±0.00%	10.00%	±0.00%	60.68%	±0.00%
gB	11.35%	±0.00%	60.68%	±0.00%	10.00%	±0.00%	60.68%	±0.00%
LI	95.40%	±0.19%	100.00%	±0.00%	84.87%	±0.21%	100.00%	±0.00%
LB	95.41%	±0.17%	100.00%	±0.00%	84.77%	±0.20%	100.00%	±0.00%
DI	95.24%	±0.21%	96.58%	±0.40%	84.58%	±0.36%	96.58%	±0.40%
DB	95.19%	±0.19%	96.58%	±0.40%	84.56%	±0.34%	96.58%	±0.40%
SI	94.54%	±0.14%	100.00%	±0.00%	84.10%	±0.23%	100.00%	±0.00%
SB	94.57%	±0.14%	100.00%	±0.00%	84.10%	±0.28%	100.00%	±0.00%
iW	98.21%	±0.09%	77.50%	±0.47%	89.18%	±0.24%	85.64%	±0.72%
ig	98.20%	±0.07%	77.66%	±0.46%	88.98%	±0.23%	85.92%	±0.41%
iL	98.14%	±0.07%	94.68%	±0.34%	88.87%	±0.26%	95.95%	±0.38%
iD	97.97%	±0.06%	98.38%	±0.42%	88.71%	±0.25%	98.37%	±0.43%
iS	98.08%	±0.09%	96.61%	±0.42%	88.90%	±0.20%	96.72%	±0.38%
igI	96.37%	±0.15%	77.38%	±0.50%	78.02%	±0.48%	77.38%	±0.50%
igB	96.45%	±0.11%	81.20%	±0.38%	79.03%	±1.14%	82.35%	±0.55%

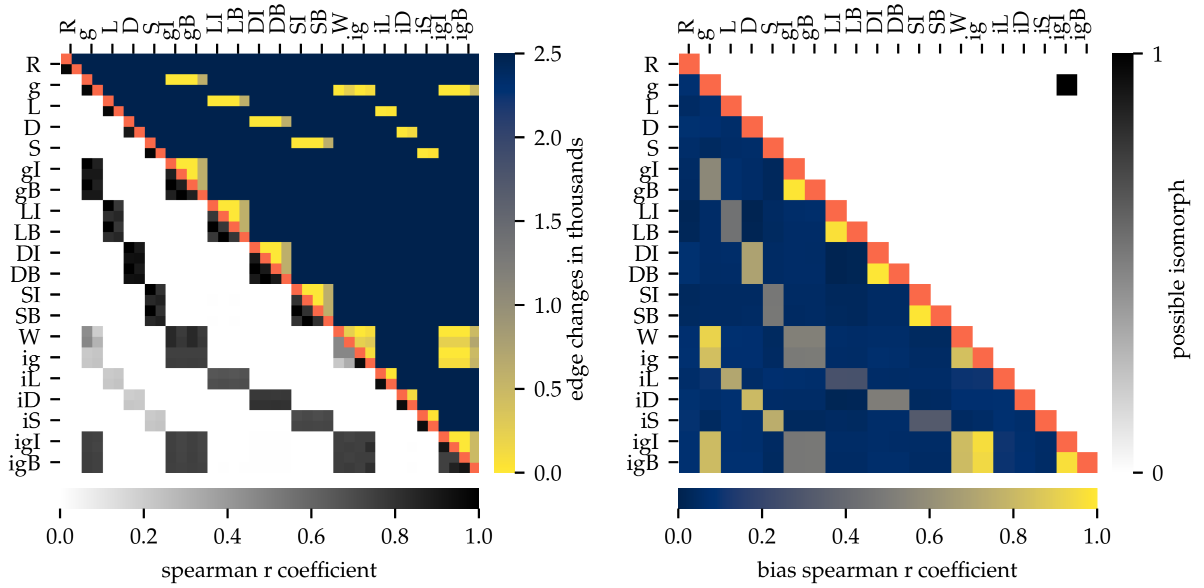

(a) Toast plot Type 1: Spearman R coefficient of weight changes (bottom left), number of edge changes (top right).

(b) Toast plot type 2: Spearman R coefficient of bias changes (bottom left), results of isomorphism test (top right).

Figure S5. Feed forward network *LeNet 300-100* mean degree 22 for *CIFAR-10*

Table S5. Comparison of best validation accuracy and fraction of the largest weakly connected component *S* for *LeNet 300-100* mean degree 22 for *CIFAR-10* dataset. ±-values show standard deviation over ten independent random iterations.

	CIFAR-10			
**ID**	**Best accuracy**		*S*	
R	53.77%	±1.22%	100.00%	±0.00%
g	53.65%	±0.68%	100.00%	±0.00%
L	54.92%	±1.55%	100.00%	±0.00%
D	53.55%	±0.82%	100.00%	±0.00%
S	53.37%	±1.39%	100.00%	±0.00%
gI	43.07%	±0.61%	100.00%	±0.00%
gB	43.32%	±0.81%	100.00%	±0.00%
LI	45.45%	±1.61%	100.00%	±0.00%
LB	45.75%	±1.54%	100.00%	±0.00%
DI	43.71%	±1.64%	100.00%	±0.00%
DB	43.65%	±1.30%	100.00%	±0.00%
SI	41.25%	±1.73%	100.00%	±0.00%
SB	41.30%	±1.57%	100.00%	±0.00%
iW	57.29%	±1.00%	100.00%	±0.00%
ig	54.97%	±1.26%	100.00%	±0.00%
iL	55.54%	±1.57%	100.00%	±0.00%
iD	55.63%	±1.14%	100.00%	±0.00%
iS	53.71%	±0.97%	100.00%	±0.00%
igI	53.14%	±1.04%	100.00%	±0.00%
igB	52.31%	±1.20%	100.00%	±0.00%

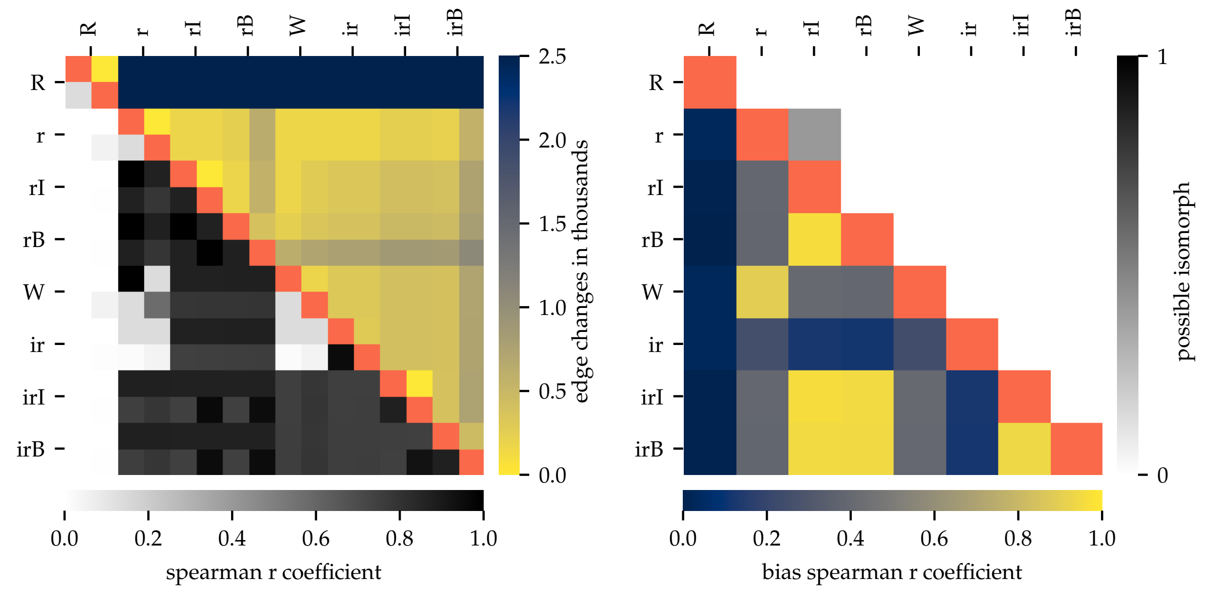

(a) Toast plot Type 1: Spearman R coefficient of weight changes (bottom left), number of edge changes (top right).

(b) Toast plot type 2: Spearman R coefficient of bias changes (bottom left), results of isomorphism test (top right).

Figure S6. Recurrent network $G(n, p)$ *3482* for *CIFAR-10*

Table S6. Comparison of best validation accuracy and fraction of the largest weakly connected component S for $G(n, p)$ *3482* for *CIFAR-10* dataset. ±-values show standard deviation over ten independent random iterations.

	CIFAR-10			
**ID**	**Best accuracy**		S	
R	49.40%	±0.94%	100.00%	±0.00%
r	49.73%	±0.63%	100.00%	±0.00%
rI	41.07%	±0.43%	100.00%	±0.00%
rB	41.03%	±0.40%	100.00%	±0.00%
iW	50.09%	±0.77%	100.00%	±0.00%
ir	51.24%	±0.59%	100.00%	±0.00%
irI	41.20%	±0.49%	100.00%	±0.00%
irB	40.98%	±0.45%	100.00%	±0.00%

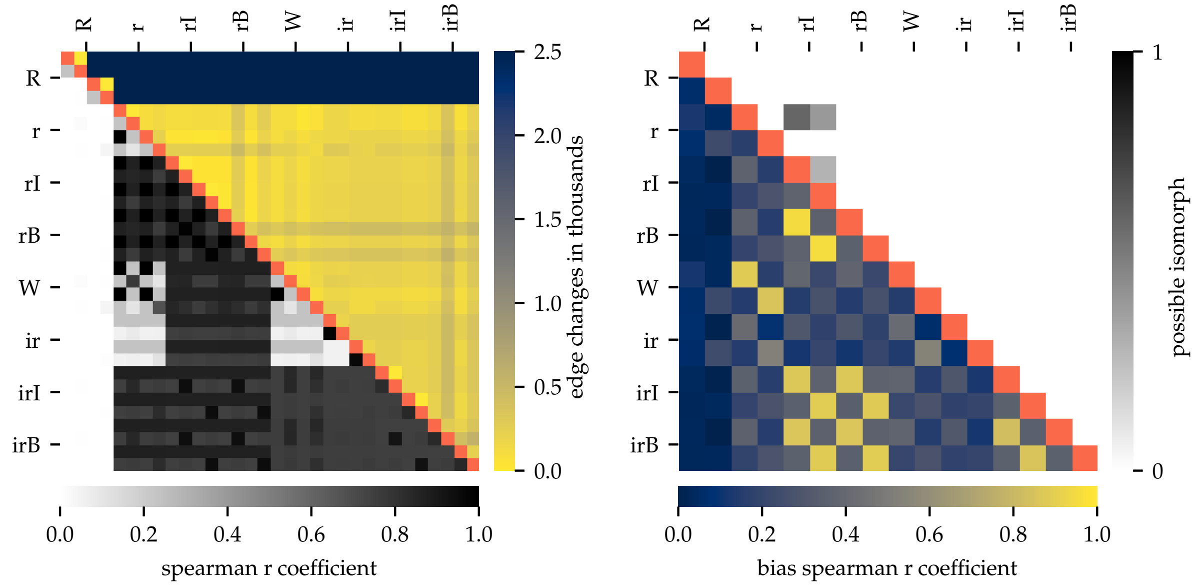

(a) Toast plot Type 1: Spearman R coefficient of weight changes (bottom left), number of edge changes (top right).

(b) Toast plot type 2: Spearman R coefficient of bias changes (bottom left), results of isomorphism test (top right).

Figure S7. Recurrent network $G(n,p)$ *1194*

Table S7. Comparison of best validation accuracy and fraction of the largest weakly connected component S for $G(n,p)$ *1194* dataset. ±-values show standard deviation over ten independent random iterations.

	MNIST				*Fashion-MNIST*			
**ID**	**Best accuracy**		S		**Best accuracy**		S	
R	97.71%	±0.15%	100.00%	±0.00%	87.69%	±0.18%	100.00%	±0.00%
r	97.70%	±0.11%	100.00%	±0.00%	87.70%	±0.38%	100.00%	±0.00%
rI	93.91%	±0.17%	100.00%	±0.00%	83.20%	±0.31%	100.00%	±0.00%
rB	93.97%	±0.22%	100.00%	±0.00%	83.21%	±0.34%	100.00%	±0.00%
iW	97.80%	±0.12%	100.00%	±0.00%	87.70%	±0.39%	100.00%	±0.00%
ir	97.76%	±0.12%	100.00%	±0.00%	88.39%	±0.23%	100.00%	±0.00%
irI	93.91%	±0.25%	100.00%	±0.00%	83.35%	±0.39%	100.00%	±0.00%
irB	94.04%	±0.23%	100.00%	±0.00%	83.25%	±0.39%	100.00%	±0.00%

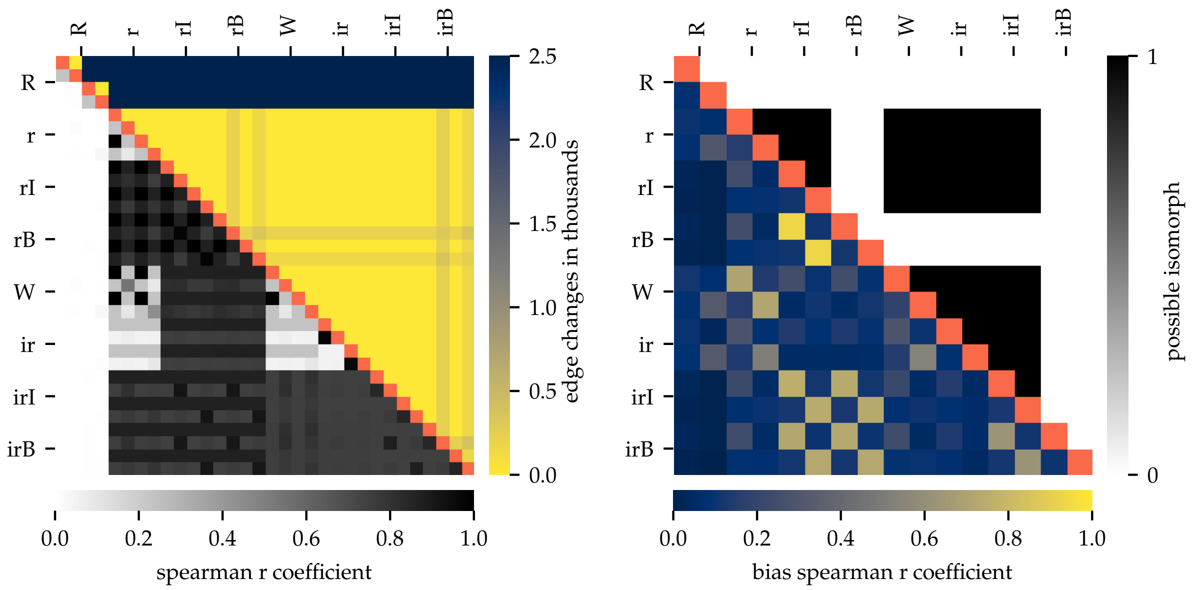

(a) Toast plot Type 1: Spearman R coefficient of weight changes (bottom left), number of edge changes (top right).

(b) Toast plot type 2: Spearman R coefficient of bias changes (bottom left), results of isomorphism test (top right).

Figure S8. Recurrent network *small world 1194*

Table S8. Comparison of best validation accuracy and fraction of the largest weakly connected component S for *small world 1194* dataset. ±-values show standard deviation over ten independent random iterations.

	MNIST				*Fashion-MNIST*			
**ID**	**Best accuracy**		S		**Best accuracy**		S	
R	98.00%	±0.07%	100.00%	±0.00%	88.32%	±0.22%	100.00%	±0.00%
r	98.00%	±0.07%	100.00%	±0.00%	88.37%	±0.40%	100.00%	±0.00%
rI	95.12%	±0.21%	100.00%	±0.00%	84.74%	±0.41%	100.00%	±0.00%
rB	95.15%	±0.15%	100.00%	±0.00%	84.85%	±0.30%	100.00%	±0.00%
iW	98.04%	±0.09%	100.00%	±0.00%	88.75%	±0.31%	100.00%	±0.00%
ir	97.90%	±0.06%	100.00%	±0.00%	88.73%	±0.30%	100.00%	±0.00%
irI	95.12%	±0.20%	100.00%	±0.00%	84.81%	±0.29%	100.00%	±0.00%
irB	95.13%	±0.18%	100.00%	±0.00%	84.94%	±0.37%	100.00%	±0.00%

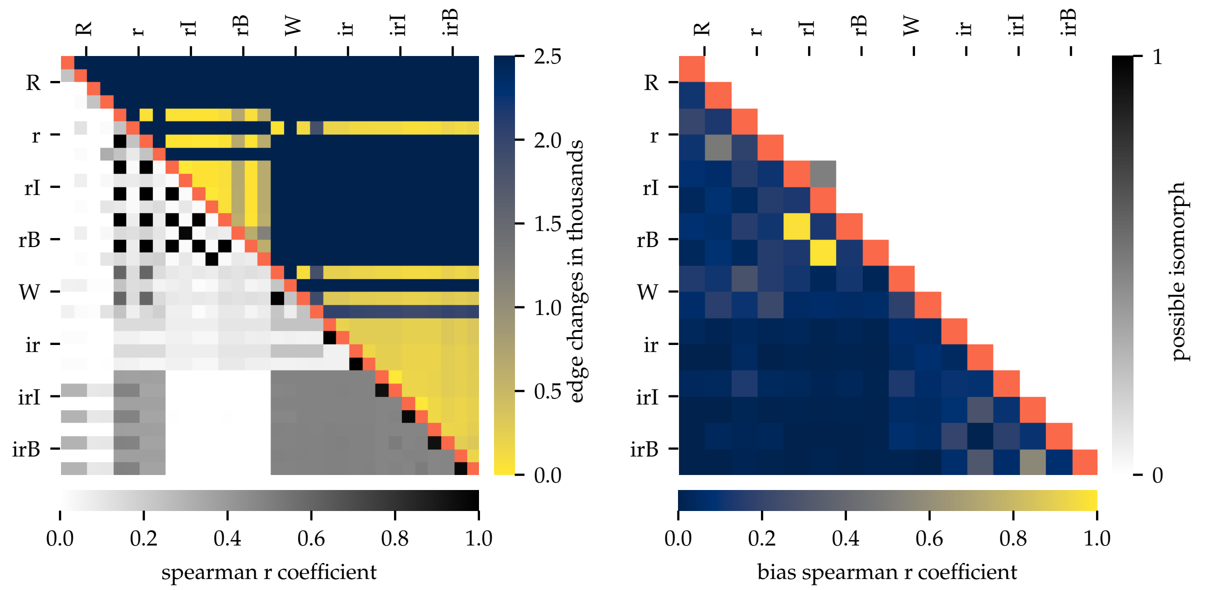

(a) Toast plot Type 1: Spearman R coefficient of weight changes (bottom left), number of edge changes (top right).

(b) Toast plot type 2: Spearman R coefficient of bias changes (bottom left), results of isomorphism test (top right).

Figure S9. Recurrent network *uniform 1194*

Table S9. Comparison of best validation accuracy and fraction of the largest weakly connected component S for *uniform 1194* dataset. ±-values show standard deviation over ten independent random iterations.

	MNIST				*Fashion-MNIST*			
**ID**	**Best accuracy**		S		**Best accuracy**		S	
R	94.61%	±0.53%	96.18%	±12.08%	85.00%	±0.31%	100.00%	±0.00%
r	94.12%	±0.45%	99.95%	±0.16%	84.96%	±0.41%	100.00%	±0.00%
rI	88.39%	±0.29%	100.00%	±0.00%	76.66%	±1.04%	100.00%	±0.00%
rB	88.42%	±0.37%	100.00%	±0.00%	76.68%	±1.05%	100.00%	±0.00%
iW	93.29%	±5.60%	96.42%	±10.22%	45.05%	±19.92%	99.96%	±0.09%
ir	20.05%	±25.97%	99.95%	±0.16%	16.30%	±21.88%	99.97%	±0.08%
irI	22.49%	±24.24%	99.97%	±0.08%	13.60%	±11.07%	99.97%	±0.11%
irB	23.76%	±23.71%	99.96%	±0.13%	12.64%	±8.77%	99.97%	±0.08%

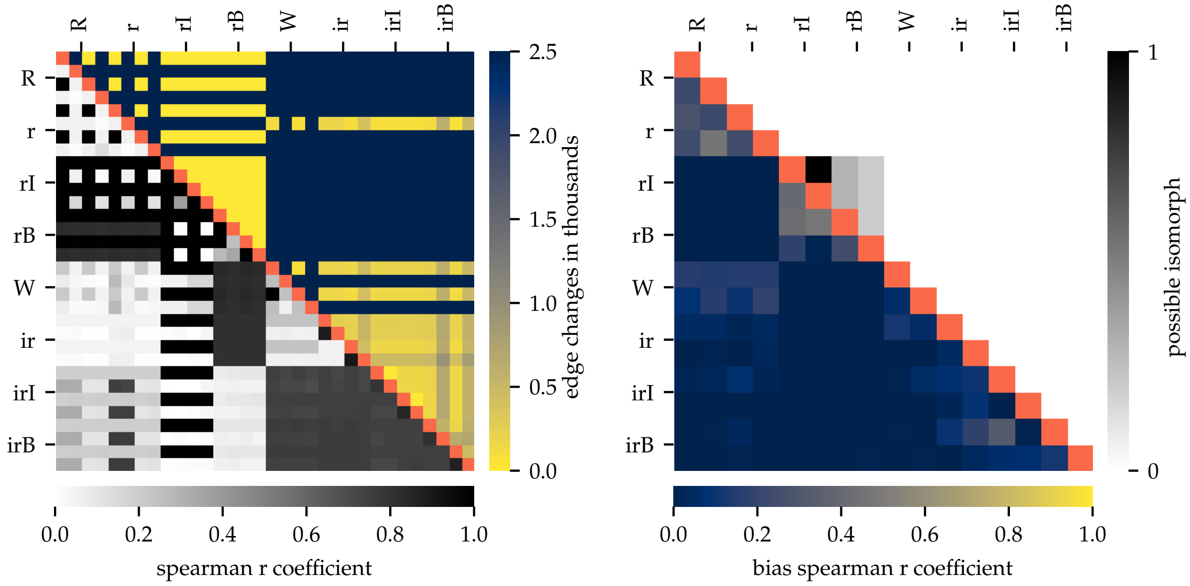

(a) Toast plot Type 1: Spearman R coefficient of weight changes (bottom left), number of edge changes (top right).

(b) Toast plot type 2: Spearman R coefficient of bias changes (bottom left), results of isomorphism test (top right).

Figure S10. Recurrent network *IRNN 1194*

Table S10. Comparison of best validation accuracy and fraction of the largest weakly connected component S for *IRNN 1194* dataset. ±-values show standard deviation over ten independent random iterations.

	MNIST				*Fashion-MNIST*			
**ID**	**Best accuracy**		S		**Best accuracy**		S	
R	95.19%	±0.51%	59.29%	±1.67%	81.13%	±1.71%	66.41%	±0.11%
r	95.11%	±0.28%	60.39%	±0.59%	81.50%	±0.96%	66.47%	±0.06%
rI	11.35%	±0.00%	0.08%	±0.00%	10.00%	±0.00%	0.08%	±0.00%
rB	14.98%	±2.98%	0.17%	±0.08%	17.14%	±4.66%	0.16%	±0.05%
iW	82.75%	±16.60%	60.67%	±0.34%	53.78%	±21.89%	62.68%	±2.14%
ir	70.89%	±38.16%	60.39%	±0.59%	23.54%	±23.28%	60.54%	±0.75%
irI	56.31%	±25.06%	63.82%	±4.86%	18.27%	±10.32%	63.61%	±4.76%
irB	58.99%	±16.91%	63.45%	±4.05%	15.70%	±5.14%	61.65%	±2.07%

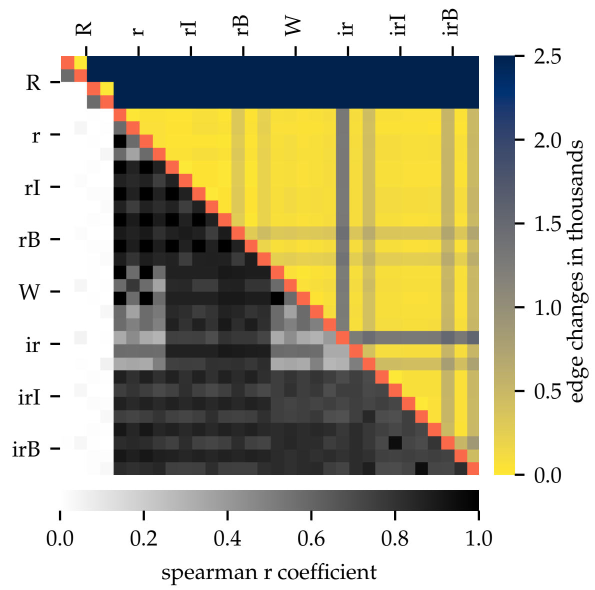

(a) Toast plot Type 1: Spearman R coefficient of weight changes (bottom left), number of edge changes (top right).

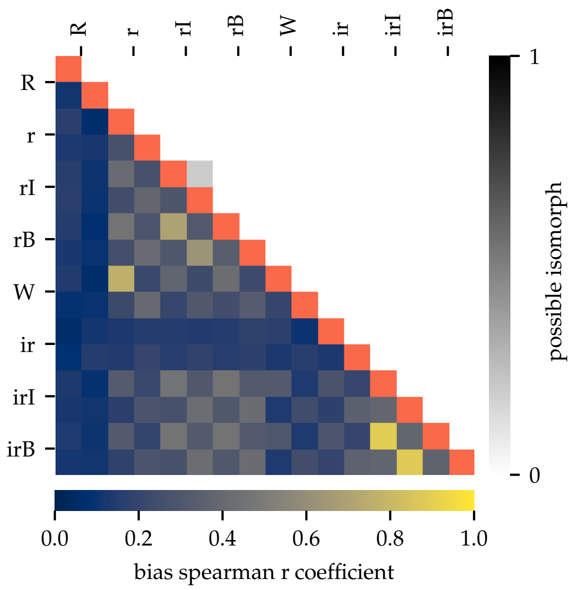

(b) Toast plot type 2: Spearman R coefficient of bias changes (bottom left), results of isomorphism test (top right).

Figure S11. Recurrent network $G(n,p)$ *177*

Table S11. Comparison of best validation accuracy and fraction of the largest weakly connected component S for $G(n,p)$ *177* dataset. ±-values show standard deviation over ten independent random iterations.

	MNIST				*Fashion-MNIST*			
**ID**	**Best accuracy**		S		**Best accuracy**		S	
R	94.03%	±0.24%	100.00%	±0.00%	83.19%	±0.72%	100.00%	±0.00%
r	93.89%	±0.31%	100.00%	±0.00%	83.71%	±0.35%	100.00%	±0.00%
rI	87.61%	±0.46%	100.00%	±0.00%	77.49%	±1.31%	100.00%	±0.00%
rB	87.98%	±0.81%	100.00%	±0.00%	78.13%	±1.30%	100.00%	±0.00%
iW	94.29%	±0.13%	100.00%	±0.00%	83.63%	±0.30%	100.00%	±0.00%
ir	91.40%	±1.29%	99.94%	±0.18%	81.86%	±0.78%	100.00%	±0.00%
irI	89.44%	±1.22%	100.00%	±0.00%	77.67%	±1.22%	100.00%	±0.00%
irB	89.90%	±0.36%	100.00%	±0.00%	77.76%	±1.36%	100.00%	±0.00%

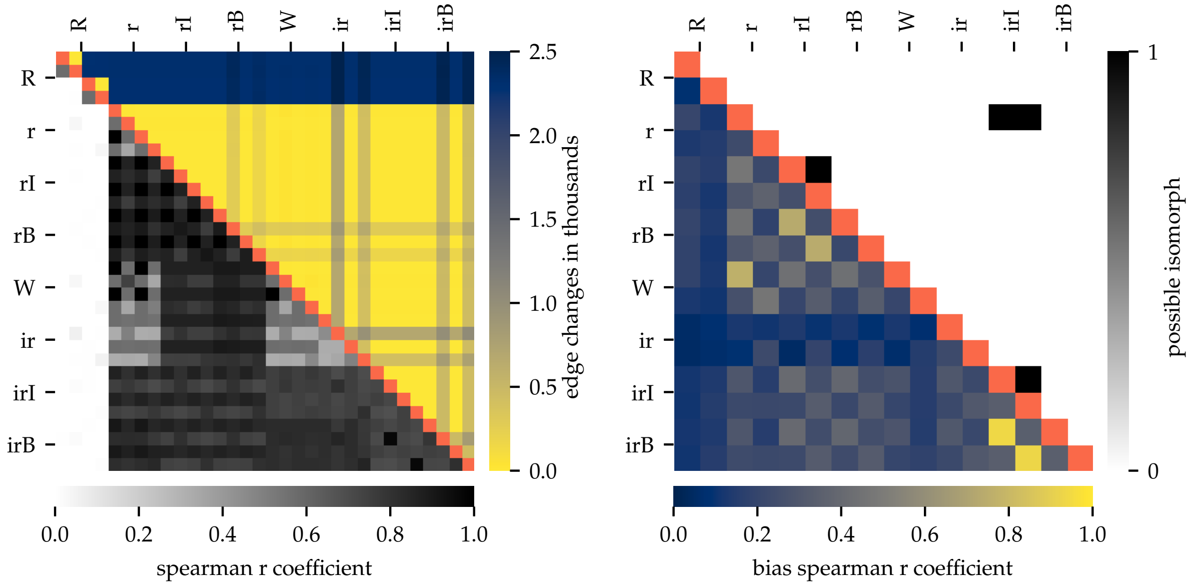

(a) Toast plot Type 1: Spearman R coefficient of weight changes (bottom left), number of edge changes (top right).

(b) Toast plot type 2: Spearman R coefficient of bias changes (bottom left), results of isomorphism test (top right).

Figure S12. Recurrent network *small world 177*

Table S12. Comparison of best validation accuracy and fraction of the largest weakly connected component S for *small world 177* dataset. ±-values show standard deviation over ten independent random iterations.

	MNIST				*Fashion-MNIST*			
**ID**	**Best accuracy**		S		**Best accuracy**		S	
R	94.08%	±0.27%	100.00%	±0.00%	83.70%	±0.52%	100.00%	±0.00%
r	94.03%	±0.15%	100.00%	±0.00%	83.63%	±0.42%	100.00%	±0.00%
rI	86.35%	±1.30%	100.00%	±0.00%	76.64%	±3.79%	100.00%	±0.00%
rB	86.89%	±2.64%	100.00%	±0.00%	77.02%	±3.01%	100.00%	±0.00%
iW	94.35%	±0.20%	100.00%	±0.00%	84.18%	±0.42%	100.00%	±0.00%
ir	91.64%	±0.95%	100.00%	±0.00%	81.92%	±0.92%	100.00%	±0.00%
irI	87.74%	±2.58%	100.00%	±0.00%	74.35%	±2.71%	100.00%	±0.00%
irB	88.25%	±2.32%	100.00%	±0.00%	73.80%	±2.80%	100.00%	±0.00%

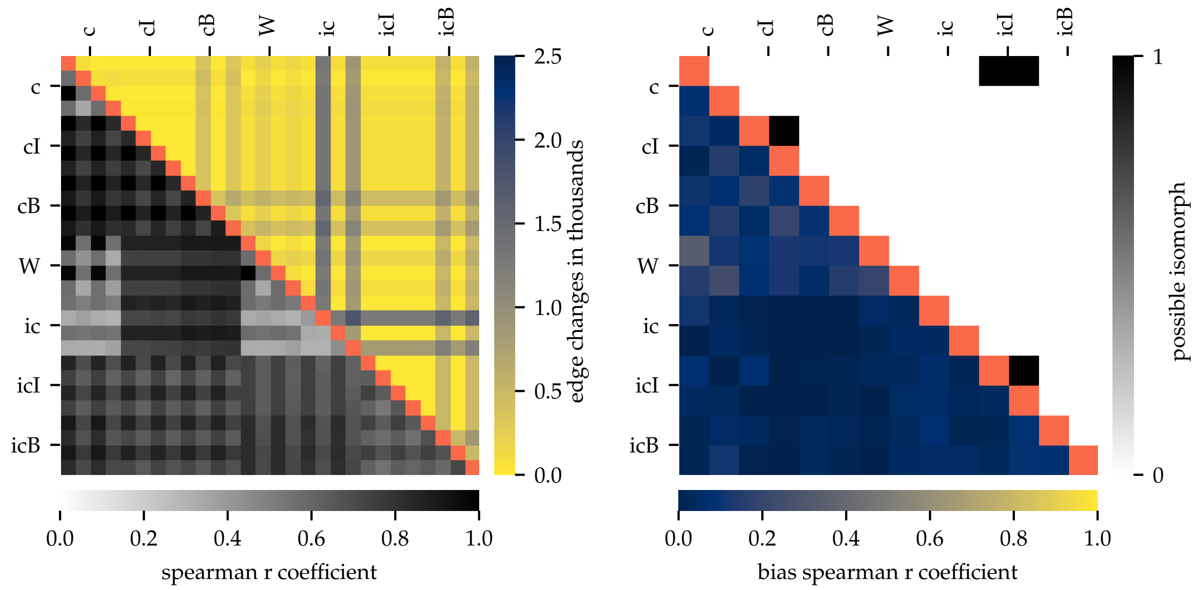

(a) Toast plot Type 1: Spearman R coefficient of weight changes (bottom left), number of edge changes (top right).

(b) Toast plot type 2: Spearman R coefficient of bias changes (bottom left), results of isomorphism test (top right).

Figure S13. Recurrent network *connectome*

Table S13. Comparison of best validation accuracy and fraction of the largest weakly connected component S for *connectome* dataset. ±-values show standard deviation over ten independent random iterations.

	MNIST				*Fashion-MNIST*			
**ID**	**Best accuracy**		S		**Best accuracy**		S	
c	93.99%	±0.50%	99.77%	±0.40%	84.26%	±0.45%	99.66%	±0.61%
cI	85.85%	±1.05%	99.66%	±0.61%	78.87%	±1.07%	99.66%	±0.61%
cB	85.78%	±2.43%	100.00%	±0.00%	78.44%	±1.51%	100.00%	±0.00%
i	94.49%	±0.18%	99.89%	±0.24%	84.49%	±0.56%	99.89%	±0.24%
ic	92.57%	±2.17%	100.00%	±0.00%	82.64%	±0.85%	100.00%	±0.00%
icI	88.06%	±0.47%	99.77%	±0.40%	78.24%	±0.89%	99.77%	±0.40%
icB	87.86%	±0.82%	100.00%	±0.00%	78.20%	±0.84%	100.00%	±0.00%

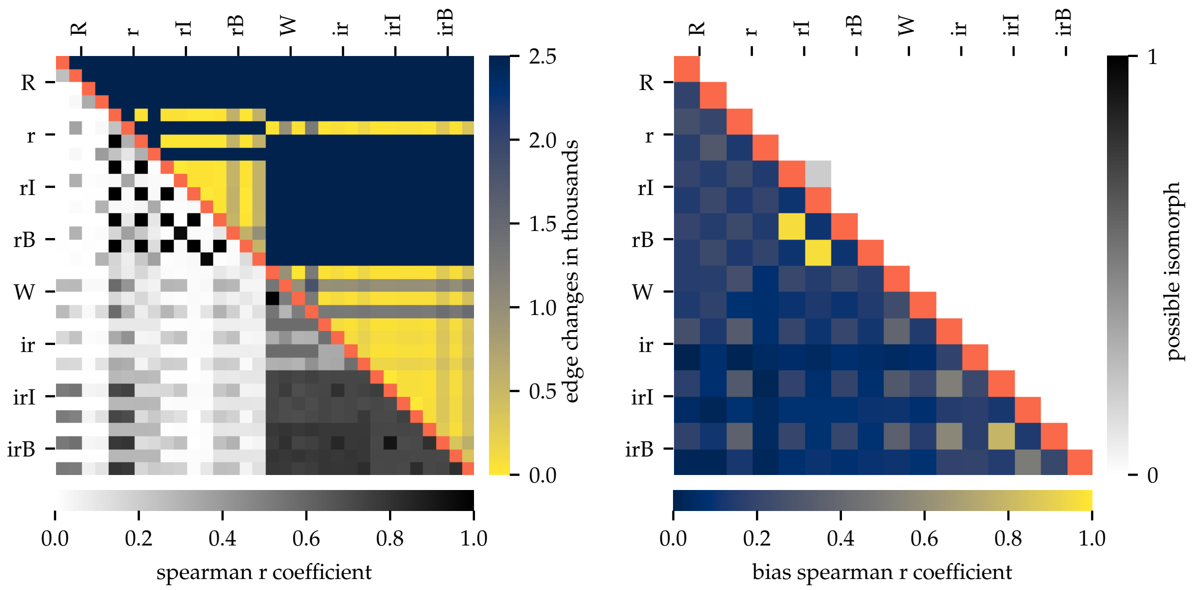

(a) Toast plot Type 1: Spearman R coefficient of weight changes (bottom left), number of edge changes (top right).

(b) Toast plot type 2: Spearman R coefficient of bias changes (bottom left), results of isomorphism test (top right).

Figure S14. Recurrent network *uniform 177*

Table S14. Comparison of best validation accuracy and fraction of the largest weakly connected component S for *uniform 177* dataset. ±-values show standard deviation over ten independent random iterations.

	MNIST				*Fashion-MNIST*			
**ID**	**Best accuracy**		S		**Best accuracy**		S	
R	91.98%	±0.83%	100.00%	±0.00%	80.91%	±1.39%	100.00%	±0.00%
r	91.53%	±1.21%	100.00%	±0.00%	81.10%	±0.75%	100.00%	±0.00%
rI	89.56%	±0.88%	100.00%	±0.00%	74.96%	±1.83%	100.00%	±0.00%
rB	89.60%	±0.41%	100.00%	±0.00%	74.48%	±2.43%	100.00%	±0.00%
iW	92.63%	±2.06%	100.00%	±0.00%	66.00%	±17.46%	99.21%	±2.31%
ir	93.80%	±0.55%	100.00%	±0.00%	66.11%	±20.19%	100.00%	±0.00%
irI	90.26%	±3.56%	100.00%	±0.00%	56.50%	±9.79%	100.00%	±0.00%
irB	90.49%	±3.56%	100.00%	±0.00%	50.47%	±11.96%	100.00%	±0.00%

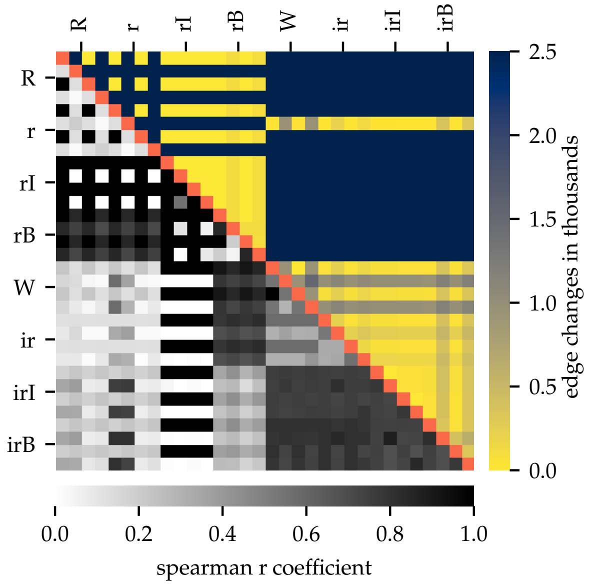

(a) Toast plot Type 1: Spearman R coefficient of weight changes (bottom left), number of edge changes (top right).

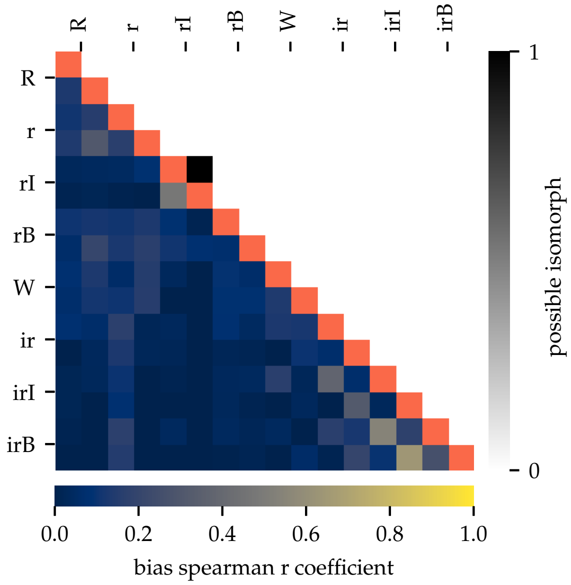

(b) Toast plot type 2: Spearman R coefficient of bias changes (bottom left), results of isomorphism test (top right).

Figure S15. Recurrent network *IRNN 177*

Table S15. Comparison of best validation accuracy and fraction of the largest weakly connected component S for *IRNN 177* dataset. ±-values show standard deviation over ten independent random iterations.

	MNIST				*Fashion-MNIST*			
**ID**	**Best accuracy**		*S*		**Best accuracy**		*S*	
R	89.37%	±1.33%	99.94%	±0.18%	77.33%	±2.31%	100.00%	±0.00%
r	89.59%	±1.19%	100.00%	±0.00%	76.51%	±0.94%	100.00%	±0.00%
rI	17.89%	±1.73%	0.56%	±0.00%	27.11%	±0.40%	0.56%	±0.00%
rB	37.52%	±4.54%	44.12%	±26.65%	44.95%	±7.00%	41.13%	±24.55%
iW	70.24%	±31.42%	100.00%	±0.00%	70.28%	±14.19%	100.00%	±0.00%
ir	91.66%	±6.11%	100.00%	±0.00%	75.44%	±6.34%	100.00%	±0.00%
irI	79.15%	±22.80%	100.00%	±0.00%	54.86%	±23.77%	100.00%	±0.00%
irB	72.06%	±30.19%	100.00%	±0.00%	53.83%	±24.56%	100.00%	±0.00%

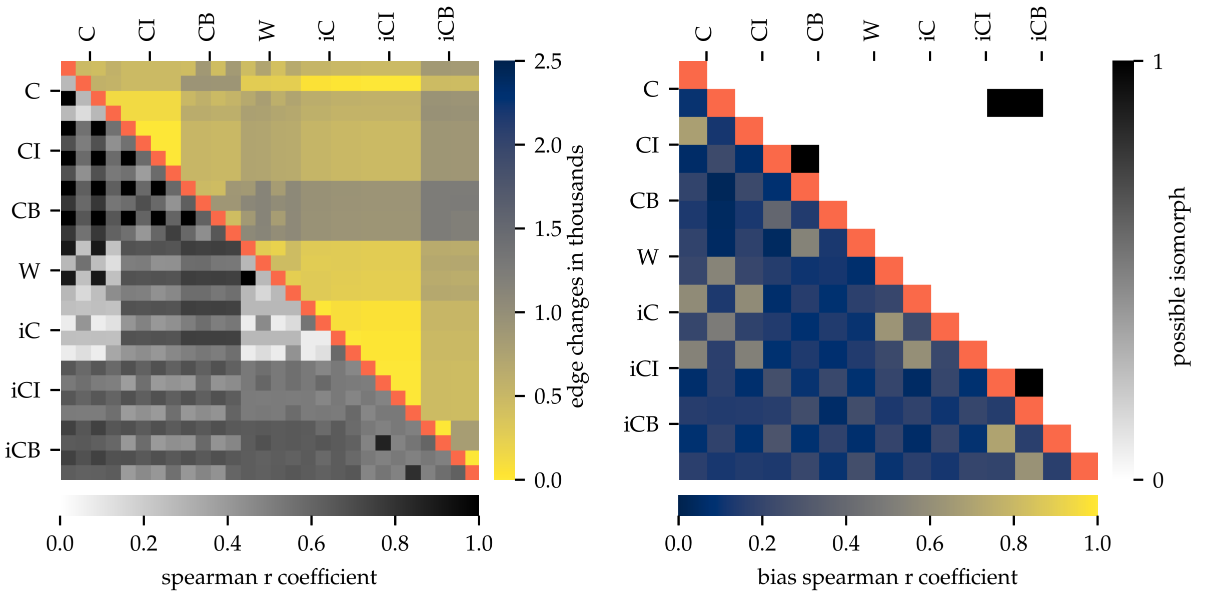

(a) Toast plot Type 1: Spearman R coefficient of weight changes (bottom left), number of edge changes (top right).

(b) Toast plot type 2: Spearman R coefficient of bias changes (bottom left), results of isomorphism test (top right).

Figure S16. Recurrent network *C. elegans*

Table S16. Comparison of best validation accuracy and fraction of the largest weakly connected component S for *C. elegans* dataset. ±-values show standard deviation over ten independent random iterations.

	MNIST				*Fashion-MNIST*			
**ID**	**Best accuracy**		S		**Best accuracy**		S	
R	95.22%	±0.24%	97.08%	±0.19%	84.87%	±0.39%	97.04%	±0.24%
C	95.05%	±0.35%	96.92%	±0.21%	84.51%	±0.55%	97.14%	±0.19%
CI	83.41%	±4.87%	96.82%	±0.00%	76.91%	±1.22%	96.82%	±0.00%
CB	85.57%	±1.87%	98.11%	±0.38%	76.34%	±2.26%	98.11%	±0.34%
iW	95.27%	±0.25%	96.92%	±0.21%	84.57%	±0.71%	96.74%	±0.53%
iC	95.46%	±0.19%	96.96%	±0.21%	84.98%	±0.24%	96.96%	±0.21%
iCI	88.57%	±0.92%	96.92%	±0.21%	77.95%	±1.70%	96.92%	±0.21%
iCB	88.87%	±1.17%	98.01%	±0.40%	77.84%	±1.62%	97.89%	±0.28%

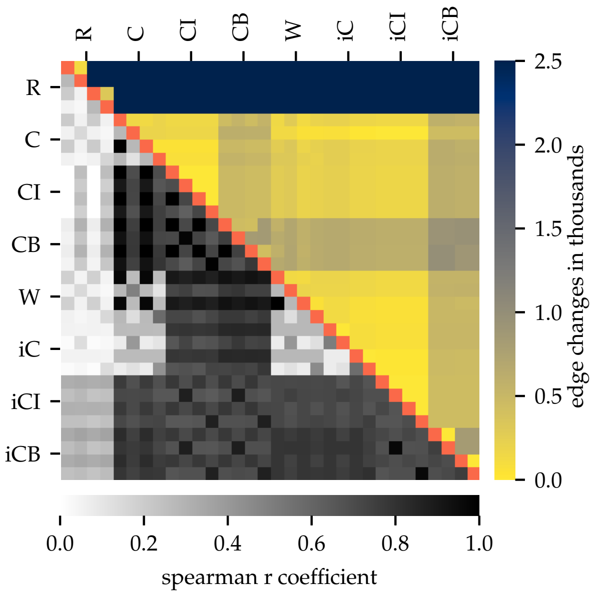

(a) Toast plot Type 1: Spearman R coefficient of weight changes (bottom left), number of edge changes (top right).

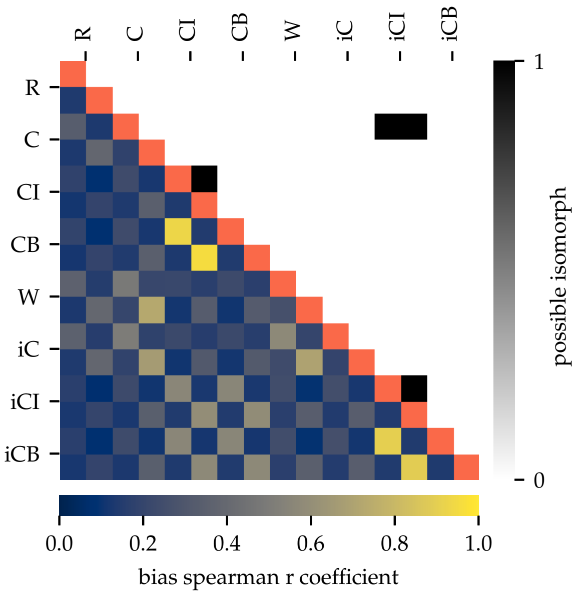

(b) Toast plot type 2: Spearman R coefficient of bias changes (bottom left), results of isomorphism test (top right).

Figure S17. Recurrent network *C. elegans* $G(n,p)$ *reference*

Table S17. Comparison of best validation accuracy and fraction of the largest weakly connected component S for *C. elegans* $G(n,p)$ *reference* dataset. ±-values show standard deviation over ten independent random iterations.

	MNIST				*Fashion-MNIST*			
**ID**	**Best accuracy**		S		**Best accuracy**		S	
R	95.10%	±0.30%	98.35%	±0.10%	83.99%	±0.85%	97.51%	±2.55%
C	94.99%	±0.37%	98.23%	±0.15%	84.45%	±0.37%	98.23%	±0.24%
CI	90.27%	±0.45%	98.41%	±0.00%	79.68%	±0.50%	98.41%	±0.00%
CB	90.29%	±0.27%	99.24%	±0.29%	79.65%	±0.51%	99.28%	±0.28%
iW	95.07%	±0.33%	98.09%	±0.14%	84.53%	±0.51%	98.19%	±0.15%
iC	95.12%	±0.28%	98.23%	±0.15%	84.79%	±0.45%	98.23%	±0.15%
iCI	90.81%	±0.28%	98.23%	±0.15%	79.99%	±0.39%	98.23%	±0.15%
iCB	90.84%	±0.38%	99.24%	±0.40%	79.93%	±0.32%	99.36%	±0.18%

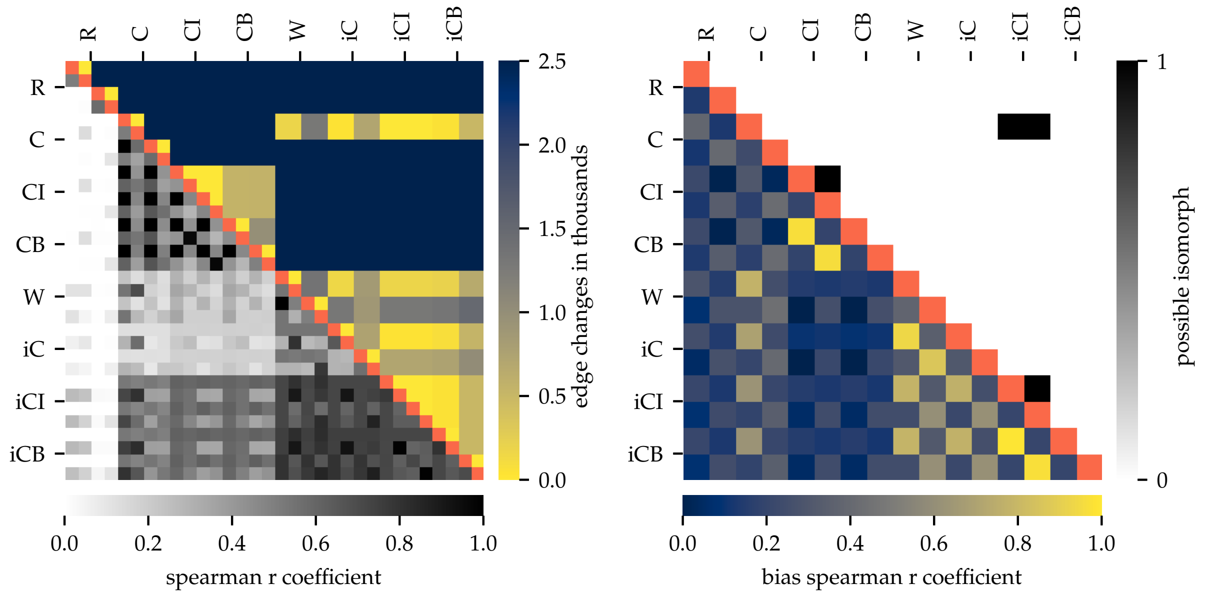

(a) Toast plot Type 1: Spearman R coefficient of weight changes (bottom left), number of edge changes (top right).

(b) Toast plot type 2: Spearman R coefficient of bias changes (bottom left), results of isomorphism test (top right).

Figure S18. Recurrent network *C. elegans LeNet reference*

Table S18. Comparison of best validation accuracy and fraction of the largest weakly connected component S for *C. elegans LeNet reference* dataset. ±-values show standard deviation over ten independent random iterations.

	MNIST				*Fashion-MNIST*			
**ID**	**Best accuracy**		S		**Best accuracy**		S	
R	92.41%	±0.17%	84.81%	±0.80%	82.38%	±0.15%	81.39%	±1.20%
C	92.31%	±0.11%	85.09%	±0.94%	82.25%	±0.15%	81.81%	±1.07%
CI	83.20%	±0.37%	95.41%	±0.20%	76.00%	±0.31%	95.41%	±0.20%
CB	83.29%	±0.43%	95.39%	±0.21%	76.07%	±0.44%	95.39%	±0.16%
iW	93.92%	±0.13%	84.85%	±1.01%	83.30%	±0.16%	85.33%	±0.79%
iC	94.33%	±0.18%	85.39%	±1.00%	83.60%	±0.18%	86.84%	±0.77%
iCI	92.31%	±0.12%	85.09%	±0.94%	80.27%	±0.34%	85.09%	±0.94%
iCB	92.32%	±0.13%	85.11%	±0.92%	80.08%	±0.31%	88.01%	±0.85%

C. elegans model schema

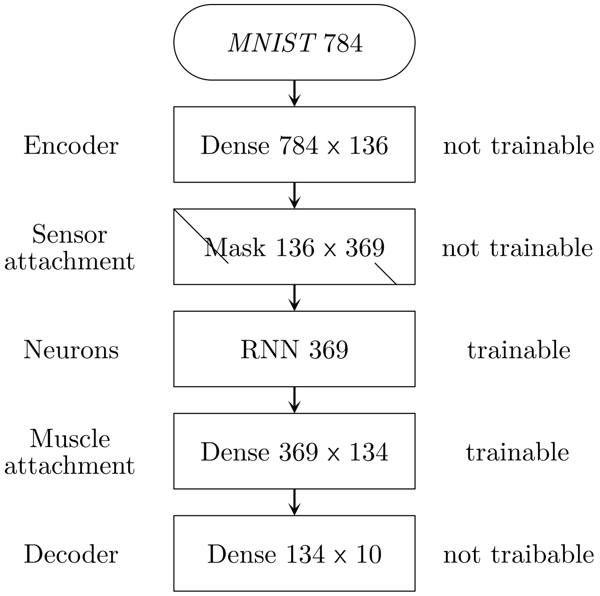

Figure S19. Simplified schematic of the *C. elegans* model. The numbers representing the dimension or the amount of neurons in the specific layer. The RNN-Layer holds the adjacency matrix of C. elengs without the muscle attachment points. The muscle attachment points are extracted to a separate dense layer to prevent that these part of the system learn recurrent connections. The mask layer distributes the encoded image to the sensor attachment points without any calculation operation. Additional supporting layers like for the time handling are not displayed.
